# Analytical approximations of the firing rate of an adaptive exponential integrate-and-fire neuron in the presence of synaptic noise

**DOI:** 10.3389/fncom.2014.00116

**Published:** 2014-09-18

**Authors:** Loreen Hertäg, Daniel Durstewitz, Nicolas Brunel

**Affiliations:** ^1^Department Theoretical Neuroscience, Bernstein-Center for Computational Neuroscience, Central Institute of Mental Health, Medical Faculty Mannheim/Heidelberg UniversityMannheim, Germany; ^2^Faculty of Science and Environment, School of Computing and Mathematics, Plymouth UniversityPlymouth, UK; ^3^Departments of Statistics and Neurobiology, University of ChicagoChicago, IL, USA

**Keywords:** adaptive exponential integrate-and-fire neuron, mean-field, Fokker-Planck equation, synaptic kinetics, spike-triggered adaptation, firing rate

## Abstract

Computational models offer a unique tool for understanding the network-dynamical mechanisms which mediate between physiological and biophysical properties, and behavioral function. A traditional challenge in computational neuroscience is, however, that simple neuronal models which can be studied analytically fail to reproduce the diversity of electrophysiological behaviors seen in real neurons, while detailed neuronal models which do reproduce such diversity are intractable analytically and computationally expensive. A number of intermediate models have been proposed whose aim is to capture the diversity of firing behaviors and spike times of real neurons while entailing the simplest possible mathematical description. One such model is the exponential integrate-and-fire neuron with spike rate adaptation (aEIF) which consists of two differential equations for the membrane potential (*V*) and an adaptation current (*w*). Despite its simplicity, it can reproduce a wide variety of physiologically observed spiking patterns, can be fit to physiological recordings quantitatively, and, once done so, is able to predict spike times on traces not used for model fitting. Here we compute the steady-state firing rate of aEIF in the presence of Gaussian synaptic noise, using two approaches. The first approach is based on the 2-dimensional Fokker-Planck equation that describes the (*V*,*w*)-probability distribution, which is solved using an expansion in the ratio between the time constants of the two variables. The second is based on the firing rate of the EIF model, which is averaged over the distribution of the *w* variable. These analytically derived closed-form expressions were tested on simulations from a large variety of model cells quantitatively fitted to *in vitro* electrophysiological recordings from pyramidal cells and interneurons. Theoretical predictions closely agreed with the firing rate of the simulated cells fed with *in-vivo*-like synaptic noise.

## 1. Introduction

In recent years there has been an increasing push toward neurobiologically highly realistic large-scale network models that incorporate a lot of anatomical and physiological detail (Traub et al., 1988; Whittington et al., 2000; Traub et al., 2005; Markram, 2006; Izhikevich and Edelman, 2008; Lansner, 2009; Lundqvist et al., 2010). One reason for this push is the growing interest in how various physiological parameters affect network dynamics, in particular in connection with pharmacologically and psychiatrically relevant questions (Markram, 2006, 2012; Kandel et al., 2013). Neural network dynamics has been identified as a crucial link between more basic genetic, molecular, and physiological factors on the one hand, and cognitive function and behavior on the other (e.g., Balaguer-Ballester et al., 2011; Mante et al., 2013, and citations therein), and has been described as a point of convergence for various pathophysiological and psychiatric mechanisms (Durstewitz and Seamans, 2012; Mitchell et al., 2012). A detailed understanding of physiological network dynamics is thus hoped to offer new insights into both how, mechanistically speaking, the healthy brain performs computations and produces behavior, and what exactly in functional and dynamical terms goes astray in psychiatric conditions. However, neurobiologically detailed models reach computational and mathematical limits very fast: Their large number of parameters, stiff non-linearities, and very high dimensionality make fitting to physiological data a very tedious *ad-hoc* process, numerical simulations very time-consuming, and prevent a deep understanding of the underlying dynamical mechanisms.

Partly for these reasons, mean-field theories (MFT) have been developed for networks of simpler single cell models such as the leaky integrate-and-fire neuron (LIF) (Abbott and van Vreeswijk, 1993; Amit and Brunel, 1997a,b; Brunel and Hakim, 1999; Fusi and Mattia, 1999; Brunel, 2000a,b; Brunel and Wang, 2001; Mattia and Del Giudice, 2002; Del Giudice et al., 2003; Renart et al., 2003, 2006; Brunel and Hansel, 2006). MFTs allow for a self-consistent mathematical description of the population dynamics on the basis of the probability density function for the membrane potential and/or other system variables (Tuckwell, 1988; Risken, 1989). Thanks to these methods, macroscopic quantities like firing rates can be derived, and various network states (e.g., asynchronous or synchronous states), together with bifurcations between these states, can be analyzed (Brunel and Hakim, 1999; Brunel, 2000a; Brunel and Hansel, 2006; Ledoux and Brunel, 2011). Furthermore, they provide a tool for systematically linking network dynamical phenomena to single cell and synaptic parameters on the one hand side, and to cognitive and behavioral observations on the other. A crucial ingredient of such MFTs is the static neuronal transfer function—how the firing rate of a single neuron depends on its mean inputs, in the presence of Gaussian noise. Such a relationship can be computed exactly for one variable such as the LIF (Siegert, 1951; Ricciardi, 1977; Amit and Tsodyks, 1991a), the exponential integrate-and-fire neuron (EIF) (Fourcaud–Trocmé et al., 2003) and the quadratic LIF (Brunel and Latham, 2003) models. Perturbative approaches have also been developed for two dimensional models, such as the LIF with colored noise (Brunel and Sergi, 1998; Moreno et al., 2002) or generalized integrate-and-fire neurons (Brunel et al., 2003).

Recent years have seen a major effort to develop very simple yet physiologically realistic single neuron models, in the sense that these can exhibit almost the full breadth of spiking patterns and bifurcations observed in real cells (Izhikevich, 2007; Naud et al., 2008; Durstewitz, 2009), and can be closely fitted in order to reproduce subthreshold voltage and spiking behavior of their empirical counterparts (Brette and Gerstner, 2005; Jolivet et al., 2006; Clopath et al., 2007; Badel et al., 2008a,b; Jolivet et al., 2008; Naud et al., 2008; Gerstner and Naud, 2009; Hertäg et al., 2012). One class of such models is the exponential integrate-and-fire neuron (Fourcaud–Trocmé et al., 2003) supplemented with an adaptation variable (AdEx, Brette and Gerstner, 2005) or a dynamic threshold variable leading to refractoriness (rEIF, Badel et al., 2008b), for which automatized, predictive and fast fitting procedures have been developed (Badel et al., 2008a,b; Hertäg et al., 2012), and which can exhibit a rich diversity of spiking dynamics due to the second variable representing spike-triggered and/or voltage-dependent adaptation (Naud et al., 2008; Touboul and Brette, 2008). Since adaptation is an almost universal feature of neuronal cells in diverse neural systems throughout the animal kingdom (Fuhrmann et al., 2002; O'Brien et al., 2002), and in cortical pyramidal neurons in particular (McCormick et al., 1985), its inclusion in spiking neuron models was an important step in reproducing a large variety of experimentally observed cell behaviors (see e.g., Izhikevich, 2007; Naud et al., 2008; Touboul and Brette, 2008; Augustin et al., 2013). Although these simple models contain a single adaptation time constant, they can often reproduce the spike adaptation behavior observed empirically (Benda and Herz, 2003; La Camera et al., 2004). While several groups have attempted to derive the firing rate of LIF-type models with an adaptation variable (La Camera et al., 2004; Gigante et al., 2007; Muller et al., 2007; Farkhooi et al., 2011), it is still unclear whether these approaches can be generalized successfully to the aEIF model, especially for parameter sets that fit electrophysiological data. Accurate analytical approximations to the firing rate of the aEIF model in such conditions would be highly desirable.

Here, we derive two approximations for the steady-state firing rate of the aEIF; first, by solving perturbatively the full 2-dimensional Fokker-Planck equation describing the aEIF with noisy inputs in the long adaptation time constant limit, and, second, by combining the 1-dimensional Fokker-Planck solution of the EIF model with distributional assumptions for the adaptation current. The theoretical ν-*I* (firing rate as a function of mean input current) curves are compared to single neuron simulations for a large number of parameter settings derived from *in-vitro* electrophysiological recordings of rodent prefrontal cortex pyramidal cells and interneurons probed with a wide range of inputs (see (Hertäg et al., 2012)). Furthermore, we investigated the influence of synaptic filtering, i.e., including realistic synaptic kinetics, through simple extensions of the Fokker-Planck formalism. These analytical expressions should provide an essential building block for the theoretical analysis of large networks composed of physiologically realistic elements, and thus improved tools for a mechanistic understanding of the relations between physiologically recorded network activity and behavior.

## 2. Results

In the following, we first derive the 2-dimensional Fokker-Planck equation for the EIF model with spike-triggered adaptation. This is a special case of the AdEx model, a simple 2-dimensional neuron model which combines the exponential integrate-and-fire (EIF) neuron (Fourcaud–Trocmé et al., 2003) with a second differential equation for an adaptation current (Benda and Herz, 2003; Izhikevich, 2003; Brette and Gerstner, 2005; Naud et al., 2008; Hertäg et al., 2012). The membrane potential *V* and the adaptation current *w* evolve according to the system of differential equations

(1) $$C\cdot \frac{dV}{dt}=-g_{L}\cdot (V-E_{L})+g_{L}\cdot \Delta_{T}\cdot e^{\left(\frac{V-V_{T}}{\Delta_{T}}\right)}+I-w$$

(2) $$\begin{array}{l} \tau_{w}\cdot \frac{dw}{dt}=a\cdot (V-E_{L})-w\\ \text{ if }V>V_{\text{up}}\text{ then }V\to V_{r}\text{ and }w\to w_{r}=w+b. \end{array}$$

Parameters *C*, *g*_*L*_, and *E*_*L*_ correspond to the capacitance, the leak conductance and the leak reversal potential of a neuron. The membrane time constant is given by τ_*m*_ = *C*/*g*_*L*_. As the membrane potential approaches the threshold *V*_*T*_, the exponential term causes a very rapid increase of voltage, modeling the fast voltage-dependent upswing of an action potential. Parameter Δ_*T*_ regulates the rise-time of the action potential. The downswing of a spike is replaced by a reset condition: When the upper threshold *V*_up_ is reached (which formally could be set to infinity), the membrane potential is reset to value *V*_*r*_, and the adaptation current is increased by a fixed step *b*, reflecting spike-triggered adaptation. In addition to spike-triggered adaptation, the AdEx model also represents subthreshold adaptation by the parameter *a*. For the sake of simplicity, we set *a* = 0 (that is, *w* depends on time only), a simplification which we previously found does not limit the model's ability to reproduce a wide range of physiological recordings and spiking patterns (Hertäg et al., 2012). For this choice of parameters, the AdEx model becomes an EIF model with firing-rate adaptation.

The second *Results* Section develops an alternative approach which incorporates into the 1-dimensional Fokker-Planck equation for the EIF model distributional assumptions for the adaptation variable *w*. The subsequent two sections then validate and compare these approaches on 156 different parameter settings obtained from electrophysiological recordings of pyramidal cells (from layers 2/3 and 5) and interneurons (bitufted and fast-spiking) from rodent PFC under *in-vivo*-like input conditions. The final *Results* Section introduces a simple method for incorporating realistic synaptic kinetics.

### 2.1. Derivation of the 2-dimensional fokker-planck equation for the aEIF model

The next step is to specify our model for the total synaptic input *I* into a cell. Cortical cells receive thousands of synaptic connections (Braitenberg and Schüz, 1991), at which transmitter release is highly probabilistic (Jahr and Stevens, 1990), and with each of them contributing only a small postsynaptic potential, on the order of 0.1–2 mV (Mason et al., 1991; Markram et al., 1997; Sjöström et al., 2001; Lefort et al., 2009; London et al., 2010). Under these conditions, the total synaptic input can be well approximated by a Gaussian stochastic process (Amit and Tsodyks, 1991a,b; Amit and Brunel, 1997a), known as the diffusion approximation limit. If one furthermore neglects the synaptic time constants, synaptic inputs take the simple form

(3) $$\frac{I(t)}{C}=\mu_{I}+\sigma_{I}\cdot \xi (t),$$

where ξ(*t*) represents a Gaussian white noise term with zero mean and unit variance, 〈 ξ(*t*) ξ (*t*′) 〉 = δ(*t* − *t*′), and the parameters μ_*I*_ and σ_*I*_ are related to the mean and standard deviation of the input current. The dynamics of the neurons as specified by Equations (1–2) provide the link between input currents and spike output. Since these equations are stochastic, the central step in relating synaptic inputs to firing rates is the derivation of equations for the dynamics of the joint probability distribution *P*(*V*, *w*) for the two dynamical variables *V* and *w* of the single-cell model. This leads to a partial differential equation known as a Fokker-Planck equation, which can be derived from the Langevin equations, (1–3) (see Models and Methods)

(4) $$\begin{array}{l} \tau_{m}\partial_{t}P(V,w)=\frac{\sigma^{2}}{2}\partial_{V}^{2}P(V,w)-\partial_{V}\left(\left[f(V)-\frac{w}{g_{L}}\right]P(V,w)\right)\\ \;+\;\partial_{w}\left[\frac{\tau_{m}}{\tau_{w}}wP(V,w)\right]. \end{array}$$

with $f(V)=-(V-E_{L})+\Delta_{T}\cdot e^{\frac{V-V_{T}}{\Delta_{T}}}+\mu_{I}\cdot \tau_{m}$ and $\sigma =\sigma_{I}\;\cdot \;\sqrt{2\tau_{m}}$.

This equation can be rewritten as a continuity equation,

(5) $$\partial_{t}P(V,w)+\partial_{V}J_{V}(V,w)+\partial_{w}J_{w}(V,w)=0$$

where *J*_*V*_(*V*, *w*) and *J*_*w*_(*V*, *w*) are probability fluxes in the *V* and *w* direction, respectively

(6) $$J_{V}(V,w)=\frac{1}{\tau_{m}}\text{​}\left(\text{​}-\frac{\sigma^{2}}{2}\partial_{V}P(V,w)+\text{​}\left[f(V)-\frac{w}{g_{L}}\right]\text{​}P(V,w)\text{​}\right)\text{​}$$

(7) $$J_{w}(V,w)=-\frac{1}{\tau_{w}}wP(V,w).$$

The boundary conditions are *P*(*V*_*up*_, *w*) = 0 for all *w*, and both fluxes should vanish sufficiently fast at *V* = −∞, and *w* = ± ∞. The steady-state firing rate ν is given by the integral of the probability flux in the *V* direction at (*V*_*up*_, *w*), integrated over all possible values of *w*,

(8) $$\nu =\int dwJ_{V}(V_{up},w).$$

There is an additional boundary condition at reset, that expresses the fact that neurons that fire at (*V*_*up*_, *w*) are reset at (*V*_*r*_, *w* + *b*): This leads to an additional flux at *V*_*r*_, such that

(9) $$J_{V}(V_{r}^{+},w+b)=J_{V}(V_{r}^{-},w+b)+J_{V}(V_{up},w).$$

**Figure 1:**
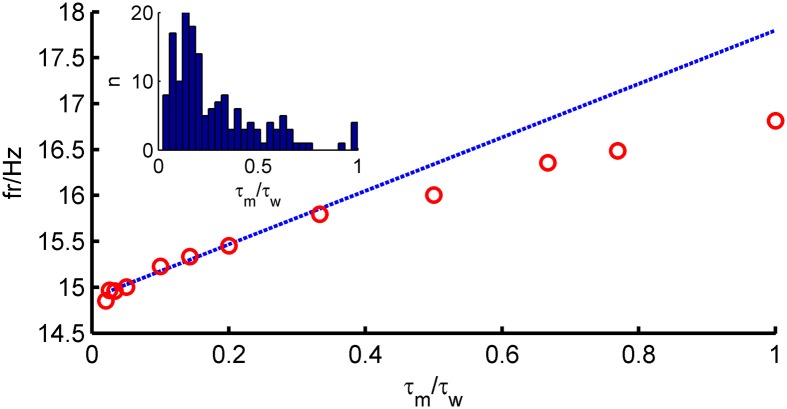
**Comparison of theoretical firing rate ν with simulation results as a function of τ_*m*_/τ_*w*_**. Model parameters for an average fast-spiking interneuron (see **Table 1**), 〈*w*〉 = *b*ν_0_τ_*w*_ constant, σ_I_syn__ = 150 pA, μ_I_syn__ = rheobase + 10 pA. Blue, theoretical prediction; red, simulation.

To solve Equation (4), we resort to a perturbative approach. In many neurons, adaptation is very slow compared to the membrane time constant (Womble and Moises, 1992; Powers et al., 1999; Benda and Herz, 2003; Stocker, 2004; Thomson and Lamy, 2007). Figure 1 (inset) shows that for most neurons in our data set, τ_*w*_ ≫ τ_*m*_ (〈 τ_*m*_ 〉 = 28 ms, 〈 τ_*w*_ 〉 = 196 ms). We therefore explore the limit τ_*w*_ ≫ τ_*m*_ and define a small parameter ϵ = $\sqrt{\tau_{m}/\tau_{w}}$. A simple calculation shows that the average of *w* is given by *b*τ_*w*_ν. Since ν is expected to be proportional to 1/τ_*m*_, we see that *b* should scale as τ_*m*_/τ_*w*_ for the average of *w* to stay finite in the limit ϵ → 0. We thus rescale ν → ν/τ_*m*_ and *b* → ϵ^2^*bg*_*L*_. In terms of these new variables, the fluctuations of *w* are expected to be proportional to ϵ *b* $\sqrt{\nu }$. We therefore define a new variable *z*, as

(10) $$w/g_{L}=b\nu +\epsilon b\sqrt{\nu }z.$$

which describes the fluctuations of *w* around its mean, in such a way as to remain of order 1 in the ϵ → 0 limit.

Performing the *w* → *z* change of variables, we find that the steady-state Fokker-Planck equation (cf. Equation 4, ∂_*t*_ *P*(*V*, *z*) = 0) is given by

(11) $$\begin{array}{l} 0=\frac{\sigma^{2}}{2}\partial_{V}^{2}P(V,z)-\partial_{V}\text{​}\left[\text{​}(f(V)-b\nu )P(V,z)\text{​}\right]+\epsilon b\sqrt{\nu }z\partial_{V}P(V,z)\\ \;+\epsilon \sqrt{\nu }\partial_{z}P(V,z)+\epsilon^{2}\partial_{z}\left[zP(V,z)\right]\text{​​} \end{array}$$

while the boundary conditions become

(12) $$P(V_{\text{up}},z)=P(V_{r}^{+},z)-P(V_{r}^{-},z)=0,$$

(13) $$R(z)=-\frac{\sigma^{2}}{2}\partial_{V}P(V,z){|}_{V=V_{\text{up}}},$$

(14) $$-\frac{\sigma^{2}}{2}\partial_{V}P(V,z){|}_{V_{r}^{-}}^{V_{r}^{+}}=R\left(z-\frac{\epsilon }{\sqrt{\nu }}\right).$$

*R*(*z*) denotes the firing rate at *z*. Next, we expand the probability density function *P*(*V*, *z*) and the firing rate ν in powers of ϵ:

(15) $$P(V,z)=\sum_{i=0}^{\infty }\epsilon^{i}\cdot P_{i}(V,z),$$

(16) $$\;\nu =\sum_{i=0}^{\infty }\epsilon^{i}\cdot \nu_{i}$$

with ν_*i*_ and *P*_*i*_ are the *i*-th order terms in the perturbation series. By inserting these series expansions into the Fokker-Planck Equation (11), and replacing $\sqrt{\nu }$ by its Taylor series (see Models and Methods), we derived a general solution for the *i*th order correction *P*_*i*_(*V*, *z*):

(17) $$P_{i}(V,z)=\frac{2}{\sigma^{2}}\int_{V}^{V_{\text{up}}}S_{i}(u,z)e^{-\frac{2}{\sigma^{2}}\int_{V}^{u}(f(x)-b\nu_{0})dx}du,$$

with *S*_*i*_ defined by the inhomogeneous terms of the FP equation. By normalizing *P*(*V*, *z*) properly such that the probability density integrates up to 1, and transforming parameters *w* and *b*, and variable ν back to their original definitions (i.e., pA and Hz respectively), we derived the zeroth order approximation for the steady-state firing rate as

(18) $$\nu_{0}={\left(\frac{2\tau_{m}}{\sigma^{2}}\int_{-\infty }^{V_{\text{up}}}dV\int_{max(V,V_{r})}^{V_{\text{up}}}e^{-\frac{2}{\sigma^{2}}\int_{V}^{u}\left(f(x)-b\nu_{0}\tau_{w}/g_{L}\right)dx}du\right)}^{-1}.$$

This is basically the firing rate of the EIF model in the presence of white noise (Fourcaud–Trocmé et al., 2003), except that the mean input μ is reduced by an amount *b*ν_0_τ_*w*_ (cf. La Camera et al., 2004; Muller et al., 2007; Farkhooi et al., 2011). Due to the symmetry of the Fokker-Planck equation in ϵ and *z*, all odd-order contributions to the firing rate must be zero, and thus the first non-vanishing term is the second order-correction (see Models and Methods for full derivation),

(19) $$\nu_{2}=\frac{1}{\tau_{m}}\cdot \frac{b{\langle z^{2}\rangle }_{0}\left[\nu_{0}KJ^{2}KQ_{0}-b{\langle z^{2}\rangle }_{0}\nu_{0}KJ^{2}Q_{0}-R_{a}{\langle z^{2}\rangle }_{0}^{2}I_{\text{R1}}\right]}{KJKQ_{0}+b^{2}{\langle z^{2}\rangle }_{0}^{2}R_{a}\cdot KJQ_{0}+b{\langle z^{2}\rangle }_{0}^{2}R_{a}/\nu_{0}^{2}-1/(2\nu_{0}^{2})}.\text{​​​}$$

where *Q*_0_ = *Q*_0_(*V*) denotes the *V*-distribution at zeroth order, the operators

(20) $$J•=\frac{2\tau_{m}}{\sigma^{2}}\int_{V}^{V_{\text{up}}}du\;e^{-\frac{2}{\sigma^{2}}\int_{V}^{u}(f(x)-b\nu_{0}\tau_{w}/g_{L})dx}•,$$

(21) $$K•=\int_{-\infty }^{V}du\;•,$$

are evaluated at the upper threshold *V*_up_, and

(22) $${\langle z^{2}\rangle }_{0}=\frac{\nu_{0}KJKQ_{0}-\frac{1}{2\nu_{0}}}{\left(\frac{1}{\nu_{0}}+b\nu_{0}KJQ_{0}\right)},\;R_{a}=\frac{KJQ_{0}}{KJKQ_{0}-\frac{1}{2\nu_{0}^{2}}},$$

(23) $$I_{\text{R1}}=bKJQ_{0}-\frac{KJKQ_{0}}{{\langle z^{2}\rangle }_{0}}+b^{2}\nu_{0}KJ^{2}Q_{0}-\frac{b\nu_{0}KJ^{2}KQ_{0}}{{\langle z^{2}\rangle }_{0}}.$$

Hence, at second order, the steady-state firing rate is given by

(24) $$\nu =\nu_{0}+\epsilon^{2}\nu_{2}.$$

The assumption τ_*m*_ ≪ τ_*w*_ (long-correlation time limit) used in the derivations above plays an important role in the prediction capability of the full-FP approach. Figure 1 shows how the firing rate depends on the ratio τ_*m*_/τ_*w*_. It shows that the prediction is very good for ϵ < 0.5, but the agreement deteriorates for larger values of τ_*m*_/τ_*w*_. For the large pool of model cells generated from *in-vitro* recordings studied here, as will be shown below, this turned out not to be a major limitation.

### 2.2. Combining the fokker-planck equation for the EIF with distributional assumptions on adaptation

A simple approximation of the firing rate of the aEIF model is to replace adaptation *w* by its mean and plug this into the Fokker-Planck solution for the EIF model [the “zeroth order” approximation ν_0_, Equation (18)]. We found that this already yields quite reasonable results, but a significant improvement can be obtained by integrating the steady-state EIF firing rate across the marginal distribution *F*(*w*) of *w*:

(25) $$\nu_{\text{aEIF}}=\int_{-\infty }^{\infty }F(w)\cdot \nu_{\text{EIF}}(\mu -w,\sigma )\;dw.$$

Note that Equation (25) explicitly neglects the correlation between *w* and *V*, and thus cannot be exact.

In order to be able to use Equation (25), one has to compute the marginal distribution *F*(*w*). For a neuron firing as a Poisson process, the distribution could be computed exactly using the methods outlined in Gilbert and Pollak (1960). However, the aEIF spiking process is different from Poisson, especially in the suprathreshold regime. Simulation results suggested that a truncated Gamma-distribution, when inserted in Equation (25), captures the firing rate reasonably well:

(26) $$\begin{array}{l} F(x,\;k,\;\theta )=\{\begin{array}{l} \frac{x^{\;k-1}\;\cdot \;e^{-x/\theta }}{\theta^{k}\;\cdot \;[\gamma (k,\;w_{\text{max}}/\Theta )-\gamma (k,\;w_{\text{min}}/\Theta )]}\\ \text{ for }w_{\text{min}}\leq x\leq w_{max}\\ 0\text{ otherwise} \end{array}\\ \text{ with }k=\frac{{\langle w\rangle }^{2}}{\sigma_{w}^{2}}\text{ and }\theta =\frac{\sigma_{w}^{2}}{\langle w\rangle }. \end{array}$$

where γ(*k*, *x*/θ) is the so-called lower incomplete gamma function, which is fully specified by the mean, 〈*w*〉, and variance, σ^2^_*w*_, of the adaptation current. These moments can be derived using the methods of Takács (1955) (see Models and Methods), yielding

(27) $$\langle w\rangle =b\nu \tau_{w},$$

(28) $$\;\sigma_{w}^{2}=\frac{b^{2}\tau_{w}\nu }{2}\left[\frac{1+\beta_{1}}{1-\beta_{1}}-2\tau_{w}\nu \right],$$

where β_1_ is the Laplace transform of the interspike interval (ISI) distribution at 1/τ_*w*_. Assuming again a Gamma-distribution for the ISIs parameterized by its mean and variance (see Models and Methods) one obtains for β_1_,

(29) $$\beta_{1}={\left(\frac{\tau_{w}}{\nu \sigma_{\text{ISI}}^{2}+\tau_{w}}\right)}^{{\left(\nu^{2}\sigma_{\text{ISI}}^{2}\right)}^{-1}}.$$

The truncation points *w*_min_ and *w*_max_ in the truncated gamma function, Equation (26), were, for simplicity, approximated by bounds of *w* in the case of a periodic spike train at frequency ν,

(30) $$w_{\text{min}}=\frac{b\cdot \text{exp}\left(-\frac{1}{\tau_{w}\cdot \nu }\right)}{1-\text{exp}\left(-\frac{1}{\tau_{w}\cdot \nu }\right)},$$

(31) $$w_{\text{max}}=\frac{b}{1-\text{exp}\left(-\frac{1}{\tau_{w}\cdot \nu }\right)}.$$

Assuming truncation points has computational advantages (considerable speed-up), and improves considerably the accuracy of the approximation in the suprathreshold range, where the spike trains get closer to periodic spike trains and therefore the distribution of *w* is close to being bounded by the values given in Equations (30)–(31).

### 2.3. Validation and comparison of the MF approaches on experimentally derived parameter sets

**Figure 2:**
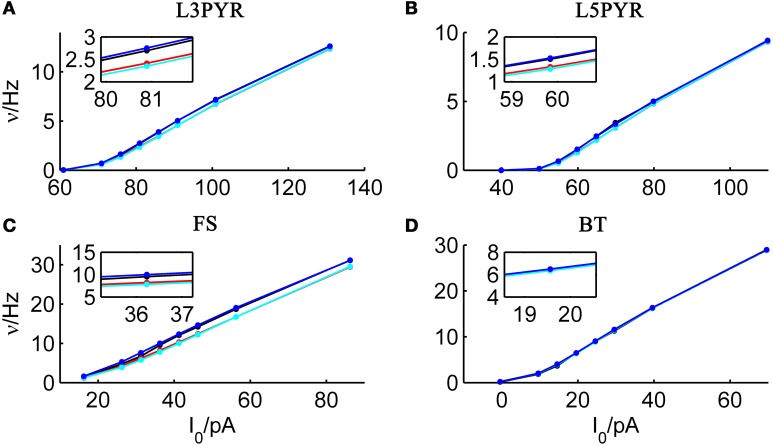
**Comparison of theoretical and simulated ν-*I* curves of the aEIF for different canonical cell types**. The ν-*I* curves for a simulated **(A)** layer-3 pyramidal cell, **(B)** layer-5 pyramidal neuron, **(C)** fast-spiking and **(D)** bitufted interneuron (averaged model parameters given in Table 1) are shown in black and compared to the theoretical predictions (ν_0_: in cyan, ν_0_ + ϵ^2^ ν_2_: in blue, 〈 ν_EIF_ 〉_*w*_: red). μ_I_syn__ ∈ {*r* − 20, *r* − 10, *r* − 5, *r*, *r* + 5, *r* + 10, *r* + 20, *r* + 50} pA with *r* denoting the cell-specific rheobase, σ_I_syn__ = 150 pA. Insets show zoom-ins on the firing rate at the rheobase.

**Figure 3:**
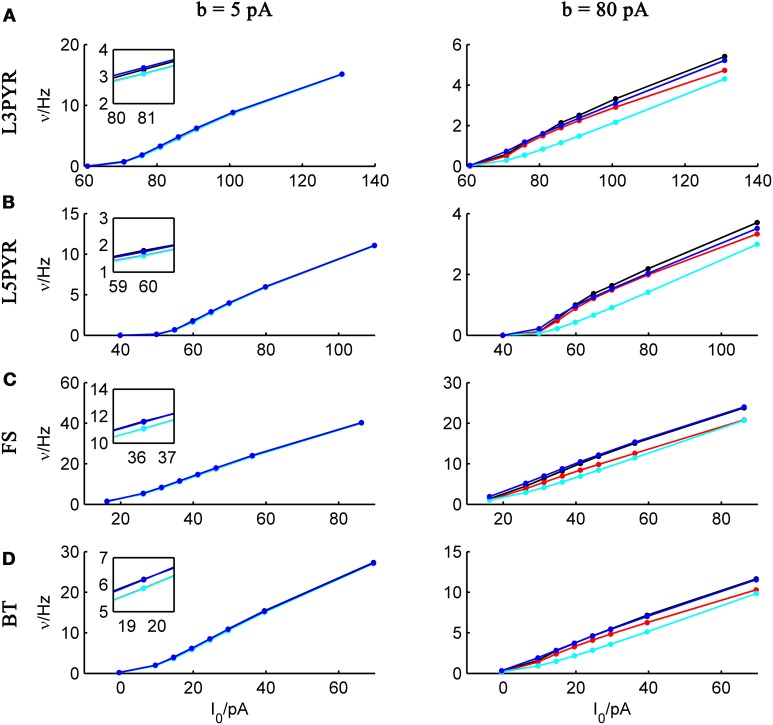
**Comparison of theoretical and simulated ν-*I* curves for small and large spike-triggered adaptation**. The ν-*I* curves for a simulated **(A)** layer-3 pyramidal cell, **(B)** layer-5 pyramidal neuron, **(C)** fast-spiking, and **(D)** bitufted interneuron (averaged model parameters given in Table 1) are shown in black and compared to the theoretical predictions (ν_0_: in cyan, ν_0_ + ϵ^2^ ν_2_: in blue, 〈 ν_EIF_ 〉_*w*_: red) for small (left panel, *b* = 5 pA) and large *b* (right panel, *b* = 80 pA). μ_I_syn__ ∈ {*r* − 20, *r* − 10, *r* − 5, *r*, *r* + 5, *r* + 10, *r* + 20, *r* + 50} pA with *r* denoting the cell-specific rheobase, σ_I_syn__ = 150 pA. Insets show zoom-ins on the firing rate at the rheobase.

**Figure 4:**
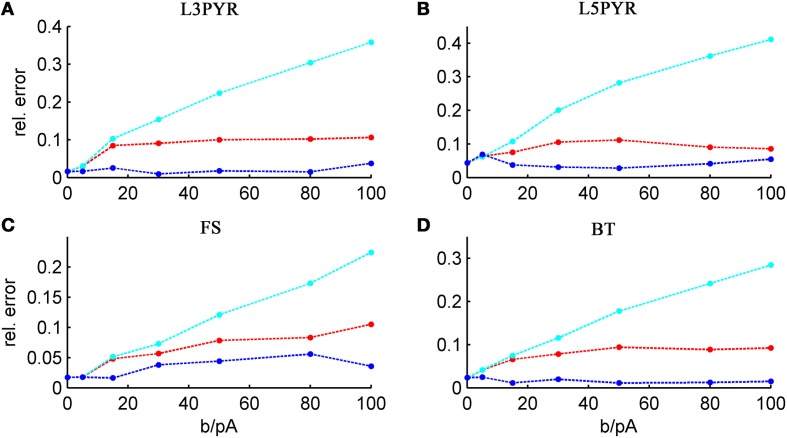
**Average relative prediction error as a function of spike-triggered adaptation for different neuron types**. For the 4 averaged neuron type parameters (**A:** layer-3 pyramidal cell, **B:** layer-5 pyramidal neuron, **C:** fast-spiking and **D:** bitufted interneuron), the average prediction error (Equation 32) is shown as a function of adaptation parameter *b* for the three theoretical approximations (ν_0_: cyan, ν_0_ + ϵ^2^ ν_2_: blue, 〈 ν_EIF_ 〉_*w*_: red). Relative prediction error was averaged across σ_I_syn__ ∈ {100, 200, 300, 400, 500, 600} pA and μ_I_syn__ = cell-specific rheobase.

We tested the three different approximations derived above (EIF-〈 w〉 approach: ν_0_, full-FP approach: ν_0_ + ϵ^2^ν_2_, EIF-F(w) approach: 〈 ν_EIF_〉_*w*_) on 4 “canonical” neocortical cell types (pyramidal cells of layers 2/3 and 5, fast-spiking, and bitufted interneurons). Parameters of these “canonical cells” were obtained by fitting a modified AdEx model (“simpAdEx,” see Hertäg et al., 2012) to a large sample from each of these 4 cell types recorded from rodent PFC slices using whole-cell patch-clamping, and taking the grand average across the estimated parameters for each cell type (averaged model parameters are given in Table 1). Figure 2 shows the input-output (ν-*I*) curves for the numerical simulations of the 4 model cells and the theoretical predictions from the three approximations (μ_*I*_syn__ ∈ [*r* − 20 pA, *r* + 50 pA] with *r* denoting the cell-specific rheobase, σ_*I*_syn__ = 150 pA). All theoretical predictions show a reasonable agreement with the simulation results. As can be seen, (1) the full-FP model tends to capture the simulation results best while the EIF-〈*w*〉 model performs worst, and (2) the largest deviation occurs for the fast-spiking interneuron, since it has the largest value of *b*, and the fastest adaptation time constant τ_*w*_. The aEIF and the EIF model with added 〈*w*〉-input differ by the fact that the latter does not account for any adaptation dynamics. Hence, we investigated more closely how the parameters governing adaptation (*b* and τ_*w*_) influence the theory-simulation agreement for the three approaches. While the adaptation time constant τ_*w*_ does not have a strong effect on the quality of the theoretical predictions, the parameter *b* which regulates the amplitude of spike-triggered adaptation plays a major role (see Figure 3). For low *b* (left panel) all three approaches do a similarly good job in predicting the simulated input-output curves, while for high *b* replacing *w* just by its average leads to serious discrepancies with the simulation results, in contrast to the other two approaches. More formally, this observation is confirmed by defining a relative goodness-of-fit measure as

(32) $${\text{Err}}_{\text{rel}}={\langle \frac{\left|\nu_{\text{sim}}-\nu_{\text{model}}\right|}{\nu_{\text{sim}}}\rangle }_{\sigma ,\;\mu }\text{ for }\nu_{\text{sim}}>0.$$

and studying its dependence on *b* starting from the four average cell configurations given in Table 1, as shown in Figure 4 (results were averaged over σ_*I*_syn__ = {100, 200, 300, 400, 500, 600} pA for a mean input current at the rheobase). Note that the non-zero error at *b* = 0 pA is due to the finite simulation time and vanishes for sufficiently long simulations. As can be seen, the goodness-of-fit steadily decreases for the EIF-〈*w*〉 model, while the relative error stays below 10% for the EIF-F(w)-based approach and below 5% for the full-FP model when *b* < 100 pA. Thus, if *b* is sufficiently small, the simplest approach of just incorporating the mean 〈*w*〉 into the 1-dimensional EIF solution seems sufficient to capture the simulated input/output relationship. For larger values of *b*, the agreement between simulations and all approaches deteriorate (see Figures 4, **6**, **7**). Interestingly, for large enough *b*, the EIF-F(w) approach outperforms the full FP approach (see **Figure 7**).

**Table 1:** **Statistics of parameter estimates of the simplified AdEx for different PFC layer 3 (L3) and 5 (L5) pyramidal cells, fast-spiking (FS) and bitufted (BT) interneurons**.

**Parameter**	**L3**	**L5**	**FS**	**BT**
*C* (pF)	125.3 [112.3] ± 62.8	246.2 [235.2] ± 86.9	48.4 [48.4] ± 13.2	80.5 [82.3] ± 28.9
*g*_*L*_ (nS)	6.0 [5.8] ± 2.5	6.9 [6.7] ± 2.2	4.3 [4.6] ± 1.0	4.3 [3.8] ± 1.3
τ_*m*_ (ms)	21.6 [19.7] ± 7.8	36.2 [33.8] ± 11.0	11.3 [11.1] ± 2.4	19.6 [19.2] ± 7.2
*E*_*L*_ (mV)	−74.6 [−73.2] ± 4.4	−71.7 [−71.2] ± 4.8	−75.5 [−74.5] ± 8.9	−79.2 [−78.5] ± 5.7
Δ_*T*_ (mV)	3.5 [3.3] ± 1.5	3.0 [2.8] ± 1.2	3.1 [3.0] ± 1.4	2.7 [2.6] ± 1.3
τ_*w*_ (ms)	229.8 [142.2] ± 213.6	263.7 [196.0] ± 198.6	25.4 [22.5] ± 11.5	74.3 [56.2] ± 64.3
*b* (pA)	37.9 [12.8] ± 49.2	25.1 [10.8] ± 35.6	66.5 [34.8] ± 104.1	30.3 [2.0] ± 111.5
*V*_*r*_ (mV)	−96.0 [−86.8] ± 36.2	−76.4 [−69.1] ± 21.7	−98.5 [−91.5] ± 29.6	−95.6 [−89.2] ± 23.8
*V*_*T*_ (mV)	−57.7 [−57.0] ± 8.1	−60.1 [−59.8] ± 7.1	−64.1 [−61.8] ± 15.2	−71.9 [−72.4] ± 8.3

Values are given as mean [median] ± SD [N(L3) = 20, N(L5) = 90, N(FS) = 25, and N(BT) = 21]. For computations, median taken for *b* and τ_*w*_ due to their long-tailed distribution.

**Figure 5:**
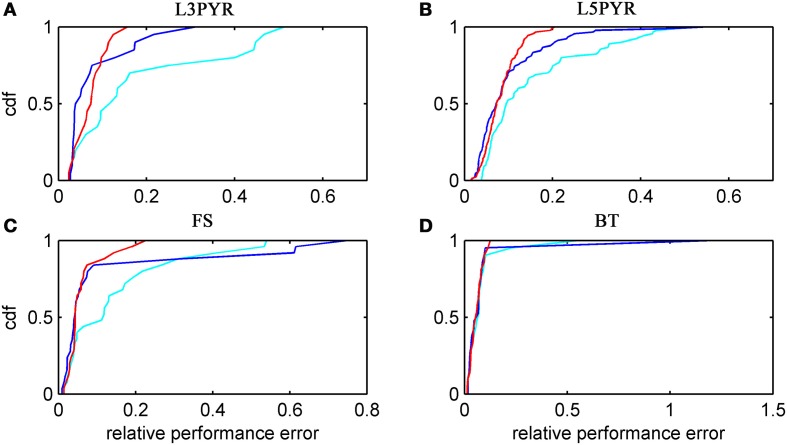
**Relative prediction error for a large physiological data pool from different neuron types recorded *in-vitro***. For the 4 neuron types (**A:** layer-3 pyramidal cell, **B:** layer-5 pyramidal neuron, **C:** fast-spiking and **D:** bitufted interneuron), the empirical cumulative distribution of the relative prediction error (Equation 32) is shown (ν_0_: cyan, ν_0_ + ϵ^2^ ν_2_: blue, 〈 ν_EIF_ 〉_*w*_: red). Relative prediction error averaged over σ_I_syn__ = (100, 300) pA and μ_I_syn__ = [*r* − 15, *r* − 10, *r* − 5, *r*, *r* + 5, *r* + 10, *r* + 15] pA with *r* denoting the cell-specific rheobase. *N*(L3) = 20, *N*(L5) = 90, *N*(FS) = 25, and *N*(BT) = 21.

**Figure 6:**
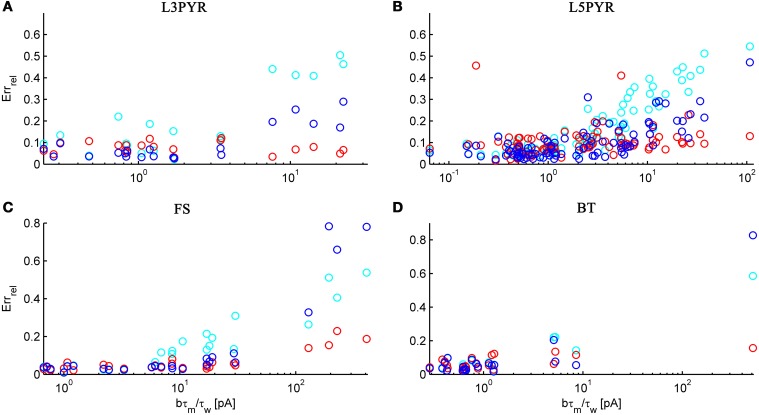
**The relative prediction error for the full FP, the EIF-〈*w*〉 and the EIF-F(w) approach as a function of *b* · τ_*m*_/τ_*w*_**. The error is shown for layer-3 pyramidal cells **(A)**, layer-5 pyramidal cells **(B)**, fast-spiking **(C)** and bitufted **(D)**. Cyan: EIF-〈*w*〉, blue: full FP, red: EIF-F(w). Pearson correlation (cyan, blue, red): (0.89, 0.86, −0.26) **(A)**, (0.74, 0.74, 0.14) **(B)**, (0.87, 0.94, 0.89) **(C)**, (0.89, 0.97, 0.51) **(D)**. Error averaged over σ_I_syn__ ∈ {100, 200, 300, 400, 500, 600} pA and μ_I_syn__ = cell-specific rheobase.

**Figure 7:**
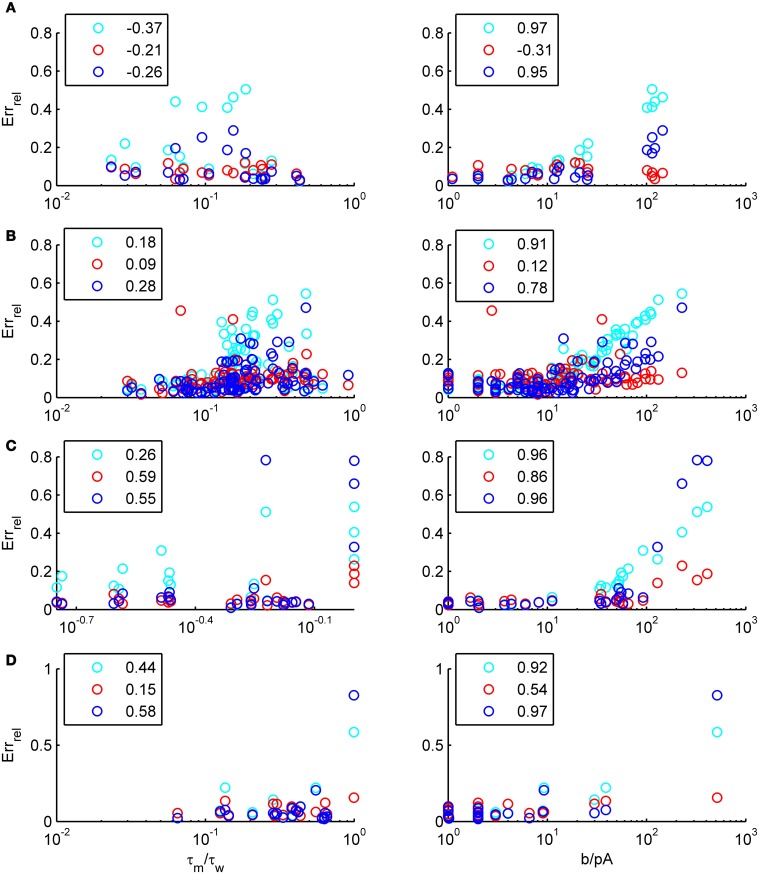
**The relative prediction error for the full FP, the EIF-〈*w*〉 and the EIF-F(w) approach as function of model parameters**. The error is shown as a function of τ_*m*_/τ_*w*_(left) and *b* (right) for layer-3 pyramidal cells **(A)**, layer-5 pyramidal cells **(B)**, fast-spiking **(C)** and bitufted **(D)**. cyan: EIF-〈*w*〉, blue: full FP, red: EIF-F(w). Pearson correlations are given in the legends. Error averaged over σ_I_syn__ ∈ {100, 200, 300, 400, 500, 600} pA and μ_I_syn__ = cell-specific rheobase.

**Figure 8:**
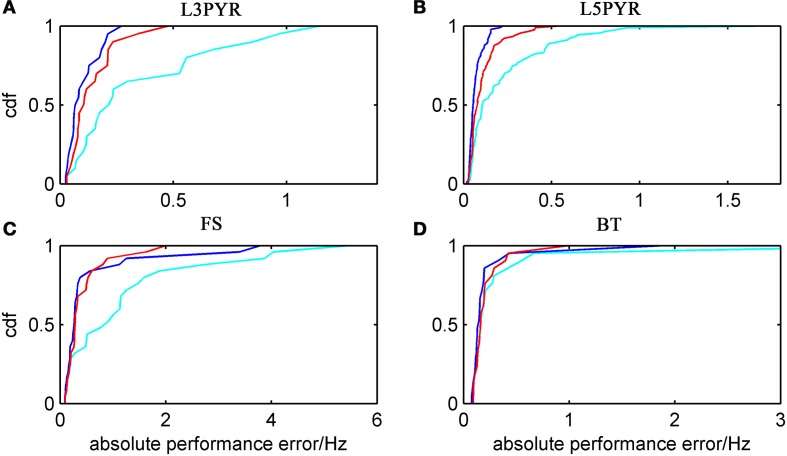
**Absolute prediction error for a large physiological data pool from different neuron types recorded *in-vitro***. For the 4 neuron types (**A:** layer-3 pyramidal cell, **B:** layer-5 pyramidal neuron, **C:** fast-spiking and **D:** bitufted interneuron), the empirical cumulative distribution of the absolute prediction error is shown (ν_0_: cyan, ν_0_ + ϵ^2^ ν_2_: blue, d〈 ν_EIF_ 〉_*w*_: red). Absolute prediction error averaged across σ_I_syn__ = [100, 300] pA and μ_I_syn__ = [*r* − 15, *r* − 10, *r* − 5, *r*, *r* + 5, *r* + 10, *r* + 15] pA with r denoting the cell-specific rheobase, and given in Hz. *N*(L3) = 20, *N*(L5) = 90, *N*(FS) = 25, and *N*(BT) = 21.

We have examined so far the accuracy of the approximations on a set of averaged model parameters representing “canonical” cortical cell types. However, even cells of the same type may strongly vary in their spiking statistics due to differences in morphological and biophysical properties, as reflected in the diversity of estimated model parameters for different cells of the same type (see Hertäg et al., 2012). To obtain a more complete picture of how the three approaches could cope with the full cellular diversity within our sample of 156 recorded rodent mPFC neurons (electrophysiological recordings and protocol were the same as in Hertäg et al., 2012, extended data set), the goodness-of-fit between simulation and theory was examined for this whole pool. Figure 5 shows the cumulative distribution of the relative prediction errors separately for each of the 4 cell classes, averaged over two input standard deviations σ_*I*_syn__ = [100, 300] pA and different mean input currents μ_*I*_syn__ = [*r* − 15, *r* − 10, *r* − 5, *r*, *r* + 5, *r* + 10, *r* + 15] pA with *r* = cell-specific rheobase. These parameters were chosen because they cover the expected range of firing rates and noise amplitudes in these cell types *in vivo*. In all four cases, the full-FP (ν_0_ + ϵ^2^ν_2_: blue) and the EIF-F(w) approximation (〈 ν_EIF_〉_*w*_: red) clearly outperform the EIF-〈*w*〉 approach (ν_0_: cyan). Interestingly, while for the cells for which the error is lowest, the full FP approach typically outperforms the EIF-F(w)-based approximation, for the cells with the largest error, the opposite happens. Hence, the EIF-F(w) approach seems to be more robust to variations in parameters. Unsurprisingly, the cells that were less well described by the analytical approximations had an uncommonly large spike-triggered adaptation parameter *b*, and/or violated the assumption τ_*w*_ ≫ τ_*m*_ (see Figures 6, 7). Note that the large majority of the cells in our sample could be captured by both the full-FP and the EIF-F(w) model with less than 10% error (full FP: 76%, EIF-F(w): 77%) while in contrast, the EIF-〈*w*〉 approach had a similar accuracy only in 54% of the cells. Thus, these results demonstrate that the best of our three approaches yields accurate results across the vast majority of empirically derived parameter settings including more than 150 recorded cells. Figure 8 shows similar results in terms of the absolute prediction error, illustrating that it rarely exceeds 1 Hz for any of the approaches. Interestingly, the full-FP approximation shows the minimal *absolute* spike rate deviation for most cells, which indicates that the largest differences occur in the low firing rate regime for this approach.

Besides empirical accuracy, an important criterion for the choice of the approach is the computation time requirements. Especially with regards to applications to large pools of data and physiologically relevant issues, the computational cost may be a decisive factor. Clearly, the EIF-〈*w*〉 approach is the fastest, but at the same time the most inaccurate. Whether taking simply the average of *w* is nevertheless still sufficient for the scientific questions at hand thus depends on the dynamical regime considered (i.e., the input regime and the spike-triggered adaptation *b*). The full-FP approach, on the other hand, provides the best results as long as the assumption of long adaptation time limit is met. However, it comes at the price of high computation time costs (due to the multi-dimensional integrals to be evaluated numerically, see Table 2). Hence, in most cases, the EIF-F(w) approximation may provide the best compromise.

**Table 2:** **Comparison of computation time requirements for the EIF-〈*w*〉, EIF-F(w), and full FP approximation**.

**Cell type**	**EIF**-〈***w*〉 approach**	**EIF-F(w) approach**	**Full FP approach**
L3	8	89	2696
L5	15	200	1740
FS	5	88	2934
BT	7	86	2096

Numbers given as mean (in seconds) for 3 typical examples of layer-3 pyramidal cells (L3), layer-5 pyramidal cells (L5), fast-spiking (FS), and bitufted (BT) interneurons. μ = cell-specific rheobase, σ = 200 pA.

### 2.4. Impact of different input regimes on theory-simulation agreement

**Figure 9:**
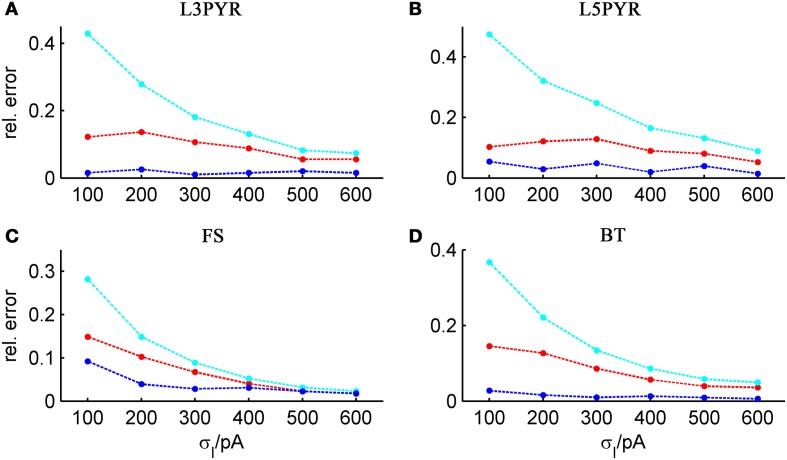
**Average relative prediction error as a function of synaptic input standard deviation σ_*I*_syn__ for different neuron types**. For the 4 averaged neuron type parameters (**A:** layer-3 pyramidal cell, **B:** layer-5 pyramidal neuron, **C:** fast-spiking and **D:** bitufted interneuron), the averaged prediction error (Equation 32) is shown in dependence on the input fluctuations σ_*I*_syn__, evaluated at the cell-specific rheobase μ_*I*_syn__, for the three derived firing rate approximations (ν_0_: cyan, ν_0_ + ϵ^2^ ν_2_: blue, 〈 ν_EIF_ 〉_*w*_: red). Relative prediction error averaged across *b* = [15, 30, 50, 80] pA.

It is widely thought that cortical cells operate in a “balanced” regime close to, but still below their spiking threshold (rheobase), with spikes caused by occasional excursions beyond the threshold driven by the fluctuations in the input (van Vreeswijk and Sompolinsky, 1996; Amit and Brunel, 1997b; Shadlen and Newsome, 1998; Brunel, 2000a; Burkitt et al., 2003; Destexhe et al., 2003; Haider et al., 2006; Renart et al., 2006; Miura et al., 2007). According to this scenario, input fluctuations play a prime role in driving cell spiking. We therefore systematically examined how the goodness-of-fit of the theoretical predictions depend on the standard deviation of the input current fluctuations. For σ_*I*_syn__ ∈ [50, 600] pA and a mean current that equals the rheobase of the cells, Table 3 shows that—depending on the examined cell class—subthreshold voltage SD's range from 2 to 12 mV, where the lower range 2–6 mV maps well onto the subthreshold voltage fluctuations observed *in-vivo* in whole cell patch-recordings as demonstrated in Hertäg et al. (2012) (see also Steriade et al., 2001; Timofeev et al., 2001; London et al., 2010). Figure 9 illustrates that the theory-simulation agreements generally improve for all three approaches as the input variance σ^2^_*I*_syn__ increases (μ_*I*_syn__ = rheobase of the respective cell). However, this tendency is particularly pronounced for the EIF-〈*w*〉 approach, while the EIF-F(w)-based and the full-FP approximations show a more steady performance across the whole range of input fluctuations. The performance gains of these models with respect to the EIF-〈*w*〉-approach are particularly large in the physiologically most relevant, *in-vivo*-like regime (i.e., for standard deviations <300 pA causing voltage fluctuation within the range observed *in vivo*, see Table 3, Hertäg et al., 2012).

**Table 3:** **Standard deviation of the subthreshold voltage fluctuations σ_*V*_ as a function of input variance σ^2^_*I*_syn__**.

**σ**_***I***__**syn**_/**pA**	**L3**	**L5**	**FS**	**BT**
50	1.52	2.42	3.54	3.38
150	2.49	2.55	4.09	3.79
250	3.47	3.01	5.39	4.59
350	4.23	3.38	6.79	5.60
500	5.62	4.21	8.96	7.41
700	7.52	5.30	11.94	9.86

Voltage fluctuations of aEIF simulations for an average pyramidal layer-3 cell (L3), layer-5 pyramidal cell (L5), fast-spiking (FS), and bitufted (BT) interneuron. Mean synaptic input μ_*I*_syn__ = rheobase of the respective cell. Spikes were cut out in a window ± 10 ms around a spike. Values given in mV.

### 2.5. Replacing the white-noise assumption by realistic synaptic kinetics

In settling on Gaussian white noise for the input process we basically assumed synaptic inputs to be delta-like current pulses. While this may be a reasonable simplification for fast (compared to the membrane time constant) AMPA- and GABA_A_-mediated currents, it is certainly not met for longer lasting currents like NMDA- or GABA_B_-mediated currents. This means our white noise model should be replaced by a colored noise model. Following Brunel et al. (2003); Alijani and Richardson (2011), steady-state firing rate correction terms may be derived that take the non-zero synaptic time constant explicitly into account. However, this would mean studying a 3-D Fokker-Planck equation, performing an expansion in two small parameters (ratios of relevant time constants) and computing the first order terms in both expansions. While this approach is certainly feasible, we resorted to a simpler approach, which consists in replacing the variance in our analytical expressions by a rescaled variance that takes into account the synaptic time constant τ_*s*_. Intuitively, this may be seen by noting that τ_*s*_ mainly influences the amplitude of the fluctuations. Furthermore, Alijani and Richardson (2011) showed that the firing rate is largely independent of the time scale of filtering as long as the amplitude of the fluctuations is adjusted such that the voltage distribution stays more or less the same. This observation can be exploited for deriving a simple approximation for the colored noise scenario. In brief, assuming that the second-order firing rate correction does not considerably change the general shape of the voltage distribution, one can derive the voltage-variance in the colored and the white noise case and deduce from their ratio a synaptic-time-constant-dependent reduction factor. For the sake of simplicity, we ignored the exponential term and went back to the LIF neuron, where the reduction of the variance is given by

(33) $$\sigma_{\text{red}}^{2}=\sigma_{I_{\text{syn}}}^{2}{\left(1+k^{2}\right)}^{-1},$$

with $k=\sqrt{\tau_{s}/\tau_{m}}$. Thus, we can proceed as before with the Gaussian white noise based theory, replacing the variance by Equation (33).

**Figure 10:**
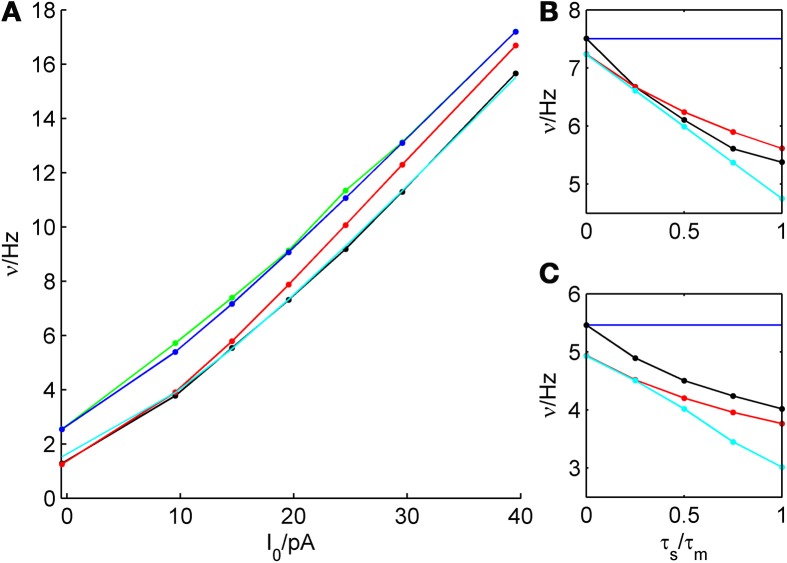
**Dependence of firing rate prediction performance on synaptic kinetics**. For an average bitufted interneuron, the theoretical predictions in the white noise and colored noise case are shown and compared to simulations. **(A):** ν-*I* curve, parameters given in Table 1, σ_I_syn__ = 250 pA, τ_*s*_/τ_*m*_ = 1/2. **(B):** σ_I_syn__ = 200 p, μ_I_syn__ = rheobase, *b* = 5 pA. **(C):** σ_I_syn__ = 200 pA, μ_I_syn__ = rheobase, *b* = 30 pA. Green: simulation, white noise; blue: theoretical prediction, white noise; black: simulation, colored noise; red: theoretical prediction (reduction of variance), colored noise. cyan: theoretical prediction by Equation (34), colored noise.

Figure 10 illustrates that the theoretically predicted and the simulated input/output curves indeed agree remarkably well when accounting for colored noise by the simple modification above, for small and large settings of *b* (Figures 10B,C). As one can see in panel (**A**), this agreement decreases in the supra-rheobase regime. Apparently, here the approach laid out above cannot fully account for the changes in *P*(*V*, *w*) brought about by the synaptic kinetics. The simple approach, Equation (33), was also compared to a more thorough theoretical derivation which explicitly corrects steady-state rates for the synaptic dynamics. For this purpose, the spike rate was expanded in powers of the ratio between the synaptic and membrane time constant *k* (see Alijani and Richardson, 2011, or Models and Methods), and in order to account for pronounced spike-triggered adaptation, the average over the theoretical *w*-distribution was taken:

(34) $$\nu_{\text{aEIF},\text{CN}}=\int_{-\infty }^{\infty }F(w)\cdot \left[\nu_{0}(\mu -w,\sigma )+k^{2}\nu_{\text{2},\text{CN}}\right]dw,$$

with the second order correction ν_2, CN_ given by Alijani and Richardson (2011)

(35) $$\nu_{\text{2},\text{CN}}=\nu_{0}\int_{-\infty }^{\infty }dV\;\int_{V}^{\infty }du\;e^{-\frac{2}{\sigma^{2}}\int_{V}^{u}f(x)dx}\left[\frac{Q_{0}}{\Delta_{T}}+\partial_{u}Q_{0}\right]\partial_{u}f,$$

where $f=-(V-E_{L})+\Delta_{T}e^{\frac{V-V_{T}}{\Delta_{T}}}-b\tau_{w}\nu_{\text{2},\text{CN}}/g_{L}$, and *Q*_0_ is the zeroth-order *V*-distribution. This method allows to reliably predict the firing rates in the sub- and supra-rheobase regime (see Figure 10, cyan line) as long as *k* ≪ 1. However, the computation of the integrals is very time-consuming such that the reduction of the variance (Equation 33) might be more suitable in terms of practicability.

## 3. Discussion

Any hope for gaining a detailed understanding of the relations between the biophysical and physiological parameters of a neural system and its emerging dynamics is ultimately encased by the computational (in-)tractability of large complex non-linear dynamical systems. Especially in the context of psychiatric disease and pharmacological intervention, there is an increasing desire to harvest computational neuroscience toward obtaining mechanistic insight (Markram, 2012; Montague et al., 2012; Kandel et al., 2013; Spanagel et al., 2013), yet the current possibilities for this are limited: Either the models are simple enough to allow systematic formal treatment and analysis (e.g., Brunel, 2000a; Brunel and Latham, 2003), but then they are often too remote from physiological reality to enable an understanding of how specific biophysical or physiological factors contribute to the system's functional dynamics. Or the models are biophysically and anatomically sophisticated (Traub et al., 1988; Whittington et al., 2000; Traub et al., 2005; Markram, 2006; Durstewitz and Gabriel, 2007; Izhikevich and Edelman, 2008; Lansner, 2009; Lundqvist et al., 2010), but suffer from tedious and time-consuming parameter estimation, long simulation times, hence limited possibilities for scanning large parameter spaces, and little hope for a systematic investigation of the system dynamics. Recently developed single neuron models, like the AdEx model, start to fill in this important gap: On the one hand, they are simple enough (2 differential equations) to enable detailed investigation of their phase space and bifurcations, as well as fast and efficient parameter estimation procedures (Badel et al., 2008a,b; Hertäg et al., 2012). On the other hand, they are sufficiently powerful to model a wide range of physiologically observed spiking patterns (Naud et al., 2008; Touboul, 2008; Touboul and Brette, 2008; Durstewitz, 2009). In fact, they can be fitted to faithfully reproduce subthreshold voltage and spiking dynamics, and even predict spike times on test sets highly dissimilar from the training set within the bounds of physiological reliability (Hertäg et al., 2012). Thus, although these models, unlike Hodgkin-Huxley models, do not explicitly include the full range of biophysical-ionic mechanisms with which real cells are equipped, they may nevertheless implicitly account for them to the degree these are reflected in the probed subthreshold and spike output dynamics. In this sense, models like the AdEx still allow for a phenomenological access to, and study, of a cell's biophysical repertoire.

Here, we developed analytical approximations to the steady-state firing rate of a population of aEIF cells under *in-vivo*-like synaptic bombardment, characterized by its Gaussian mean and variance. This ‘static transfer function’ provides the central building block for mean-field descriptions of the firing rate of different neural populations in asynchronous network states. These descriptions yield a set of self-consistent, closed-form expressions for these population firing rates. They have been highly successful in gaining a deep understanding of neural system dynamics (e.g., Amit and Brunel, 1997a; Brunel and Hakim, 1999; Brunel, 2000b; Renart et al., 2003), and in efficiently determining suitable parameters for networks of spiking neurons (Deco et al., 2009). We have extended this already established framework to the static transfer function of a neuron model of intermediate mathematical complexity yet high physiological validity, the exponential integrate-and-fire neuron with spike-triggered adaptation (aEIF). Three approaches were followed toward this goal: First, we derived the 2-dimensional Fokker-Planck (FP) equation for the full aEIF system (“full-FP approach”) and solved it under the assumption of slow (compared to the membrane time constant) adaptation, a reasonable account for most cortical neurons. Second, we replaced the adaptation variable *w* simply by its mean and inserted this into the previously derived solution of the Fokker-Planck equation for the exponential integrate-and-fire neuron (“EIF-〈*w*〉-approach”). Third, we augmented the EIF solution by accounting for the marginal distribution of *w* (“EIF-F(w)-approach”) which was derived empirically from simulation studies. The validity of the theoretical predictions was then checked on a large range of physiological parameter sets derived from ~150 *in-vitro* recordings of prefrontal cortical pyramidal cells and interneurons. While all three theoretical approaches agreed reasonably well with the numerical results for most neurons from this set, the theoretical predictions from the EIF-〈*w*〉-approach were clearly inferior to the other two MF approaches when adaptation was strong or input fluctuations were relatively low, i.e., within a range inferred from *in-vivo* patch-clamp recordings. In practice, the computation of the second-order correction for the full-FP takes the longest time due to the multi-dimensional integrals to be evaluated numerically. Therefore, the EIF-F(w) approach presents a good balance between computation time requirements and the accuracy of the firing rate agreement. It yields theoretical predictions with at most 10% of error in our large sample, usually below 2%, and furthermore is amenable to an efficient computational implementation. Finally, realistic synaptic kinetics (“colored noise”) could be incorporated by simply reducing the input variance by a theoretically derived factor that depends on the ratio of the synaptic to the membrane time constant. Thus, we have developed an approach that provides (semi-)analytical access to the dynamics of large networks composed of physiologically realistic neuron models which can be tightly fitted to electrophysiological recordings.

### 3.1. Assumptions and limitations of the mean-field approaches

**Figure 11:**
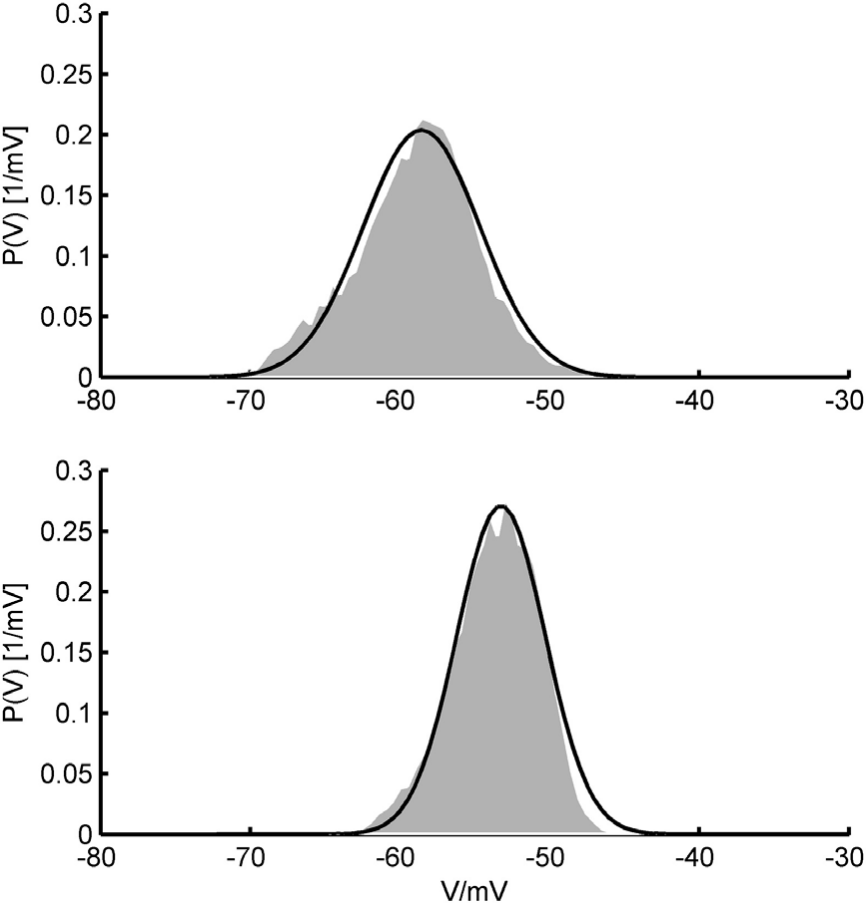
***In-vivo V*-distributions and theoretical predictions**. Two examples for the agreement of theoretically predicted membrane potential distributions (black curves) with ones obtained from *in-vivo* patch-clamp recordings (gray areas; data kindly provided by Dr. Thomas Hahn, Central Institute of Mental Health). *In-vivo* distributions were generated from voltage recordings during up-states in anesthetized rat prefrontal cortex. Theoretical fits were obtained by allowing the mean and standard deviation of the synaptic input to vary (and aligning distributions along the *V*-axis) while keeping single cell parameters fixed. Single cell parameters were drawn from the pool of AdEx model fits to *in-vitro* recordings (see text) and held constant in the process.

All of our three approaches were based on some assumptions that require discussion. A common rationale underlying the Fokker-Planck formalism is that the system's variables can be treated in the diffusion limit where the input statistics can be accurately described by their two first moments only. This ensues in the presence of a large number of relatively small synaptic inputs where the Central Limit Theorem ensures convergence to a Gaussian distribution which is fully specified by its first two moments. Physiologically, this is a very reasonable assumption given that the number of synaptic contacts a typical cortical pyramidal cell receives is indeed very high, in the range of 10^3^–10^4^ (Braitenberg and Schüz, 1991), each of them delivering a postsynaptic potential of less than 1 mV on average (Markram et al., 1997; Tsodyks and Markram, 1997; Sjöström et al., 2001; Lefort et al., 2009; London et al., 2010). Moreover, cortical spike statistics are often close to Poisson (Shadlen and Newsome, 1998), and synaptic release itself is highly probabilistic (Dobrunz and Stevens, 1997). These assumptions could, in principle, also be experimentally tested using *in-vivo* patch-clamping (London et al., 2010, see Figure 11).

With regards to the adaption current (*w*), we assumed that (i) spike-triggered processes are the major drivers of spike train adaptation, and (ii) adaptation is relatively slow in comparison with the membrane time constant. Again, both these assumptions are physiologically highly reasonable as Ca^2+^-dependent K^+^ currents (Brown and Adams, 1980; Madison and Nicoll, 1984; Sah, 1996; Powers et al., 1999) and slowly inactivating (upon spiking, τ > 1–3 s) “persistent” Na^+^ currents (Fleidervish and Gutnick, 1996; Astman et al., 2006) are the major sources of adaptation in cortical cells. Ca^2+^ influx is a strongly spike-bound process governed mainly by high-voltage-activated Ca^2+^ and voltage-dependent NMDA currents (Schiller et al., 1995; Spruston et al., 1995; Stuart et al., 1997), and builds up almost linearly with spiking rate (Helmchen et al., 1996). It furthermore decays relatively slowly with time constants usually >100 ms (Helmchen et al., 1996; Vergara et al., 1998), and major Ca^2+^-dependent K^+^ currents like AHP also have relatively slow time constants. The EIF-〈*w*〉-approach, in addition, relies on the assumption that the amplitude *b* of adaptation is relatively small for the diffusion limit to be accurate. This is the case for some, but not all of the cortical cell types we have investigated. Thus, for a decent fraction of neurons a significant improvement is achieved by using either the full-FP equation or by integrating the EIF rate across the probability density of *w* (EIF-F(w) approach). This is especially true when input fluctuations are not large and thus fluctuations in *w* tend to have a larger impact on the neuron's spiking behavior.

If the neuron's spiking process was truly Poissonian, the *w*-density could be derived explicitly following (Gilbert and Pollak, 1960). However, because of the refractory period and the adaptation process (which causes non-vanishing auto-correlations) this assumption is not fully met for the aEIF model. This leads to strong deviations, especially in the supra-rheobase regime with its higher spiking rate, such that adaptation and refractory processes have a major influence. To circumvent this, we derived the *w*-distribution empirically from simulations and found that a truncated Gamma-distribution, parameterized by the mean and the variance of the adaptation current could predict rather well the steady-state firing rate of the aEIF. In order to compute the variance of *w*, we first derived the variance of the interspike intervals which were assumed to be Gamma-distributed, an approximation which is justified by our simulations, experimental data (Barbieri et al., 2001; Miura et al., 2007; Maimon and Assad, 2009), and theoretical studies (Ostojic, 2011). We furthermore observed that replacing the analytical, parameterized truncated Gamma distribution by the true empirical density function hardly improved the results, implying that everything can still be solved in closed-form (data not shown).

### 3.2. Comparison of the analytical approaches to simulation results

In general, all approaches agreed remarkably well with numerical simulations on model neuron parameters averaged across large sets of *in-vitro* recordings from four different major cortical cell types (pyramidal cells of layers 2/3 and 5, fast-spiking, and bitufted interneurons). For a considerable fraction of cases, the EIF-〈*w*〉-approach gave comparable results to the other two, more sophisticated approaches, due to the fact that the adaptation amplitude *b*, on average, is rather small in our pool of model cells derived from experimental recordings. If *b* is sufficiently small, fluctuations in the synaptic input dominate the spike-induced fluctuations in *w*, and hence in this case the other two approaches yield no significant improvements compared to accounting just for the mean in *w*. However, we are ultimately interested in extending the approach to physiologically realistic scenarios which capture the experimentally observed diversity in parameters, so that it is important that the approach cannot “only” capture a few “canonical” cell types, but in fact a broad range of experimentally derived input-output behaviors. Investigating prediction performance across a large pool of model cells parameterized by *in-vitro* recordings from different cell types, layers, and species (rat and mice), the full-FP and EIF-F(w) approaches not only clearly had an edge over the EIF-〈*w*〉-based MF approach (cf. Figure 5), but predicted the simulated input/output curves in almost all cases with less than 10% error, across a range of different adaptation constants *b*. The few deviations from this high prediction accuracy observed for the full-FP can be explained through the fact that the assumption of a long-adaptation time limit was violated in these instances. When the range of input standard deviations was chosen such that the evoked subthreshold membrane fluctuations are most consistent with *in-vivo* data, the EIF-F(w) approach started to clearly outperform the EIF-〈*w*〉 approach (Figure 9). Thus, to reproduce average spiking behavior under *in-vivo*-like conditions, neural adaptation implemented by *w* seems to play a significant role. We had already observed previously that the inclusion of an adaptation variable in the single cell model makes a quite pronounced difference for its prediction performance on spike times from real cell counterparts when driven with *in-vivo*-like currents (Hertäg et al., 2012). More generally, spike rate adaptation appears to be one of the most important properties in accounting for the rich diversity in real neuron's spiking patterns (see e.g., Izhikevich, 2007; Naud et al., 2008; Touboul and Brette, 2008). Omitting it, or approximating it by a constant mean, may hence profoundly alter the input/output and response behavior of the modeled neural population, and thereby stable state solutions and dynamical properties. Although this certainly has to be investigated in much more detail, these observations suggest that reverting to simpler cell models in mean-field approximations may come at a prize.

In terms of practicability, the EIF-〈*w*〉 method is much faster and easier to apply than the other two approaches. The EIF-F(w) approach may represent a reasonable balance between the fast, but least accurate EIF-〈*w*〉 approach, and the most accurate, but time-consuming full-FP method. The choice of the approach should therefore be driven by the purpose of the research, the dynamical regimes considered and/or the specific cell types studied.

### 3.4. Relation to previous work on models with adaptation

Several studies have investigated the influence of adaptation at the single neuron level (or for a population of unconnected neurons). La Camera et al. (2004) incorporated the effect of adaptation into the calculation of the mean firing rate by just taking its mean, as in our EIF-〈*w*〉-approach (see also Ladenbauer et al., 2014). While they reported a good agreement with data using this approach, we have shown here that a significant fraction of recorded cells is not well described. Muller et al. (2007) used a mixed analytical and numerical approach to study various variants of LIF neurons with firing rate adaptation (see also Chizhov and Graham, 2007; Farkhooi et al., 2011). They applied a master equation for the adaptation variable, driven by a hazard function describing the instantaneous firing probability, which was obtained from fitting simulation data. Naud and Gerstner (2012) investigated the dynamics of a spike response model with adaptation (see also Toyoizumi et al., 2009). Richardson (2009) used a numerical approach (the threshold integration method introduced in Richardson, 2007) to investigate the static and dynamical transfer functions of a generalized EIF model (GEM), that include a calcium-based adaptation variable. None of these studies addressed the scenario of LIF-type neurons with diffusive noise, beyond the mean adaptation case, as we have done here. However, some of the above mentioned approaches can be used for time-dependent inputs, a situation we have not considered.

Other authors have investigated the effects of adaptation at the network level using mean-field approaches (Treves, 1993; van Vreeswijk and Hansel, 2001; Fuhrmann et al., 2002; Gigante et al., 2007; Augustin et al., 2013; Nicola and Campbell, 2013a,b; Ladenbauer et al., 2014). These studies were either performed in the absence of noise, or replaced adaptation by its average. The calculations performed here should allow to generalize these studies to the more realistic case in which both synaptic inputs and adaptation variable exhibit fluctuations with finite variance.

### 3.4. Relation to input statistics *in vivo*

Assuming white noise input implies, by definition, that all frequencies are equally present in the power spectrum, or—put differently—that synaptic inputs are (essentially) delta-like current pulses that introduce no (or only little) filtering of the Poisson spiking input. While this may be a reasonable assumption for AMPA- and GABA_A_-mediated synaptic events with decay time constants on the order of 1–3 ms (Kandel et al., 2000), much shorter than the typical membrane time constant of 10–20 ms (Kandel et al., 2000), for NMDA- and GABA_B_ currents it is certainly not met. Yet, NMDA receptor channels are major synaptic charge carriers in neocortex (Spruston et al., 1995), and other receptors with slower kinetics like GABA_B_ or cholinergic ACh receptors may significantly contribute as well (Connors et al., 1988; Deisz et al., 1997; Xiang et al., 1998). Thus, we considered colored (filtered) noise to allow synaptic inputs to have arbitrary decay time constants. Following Alijani and Richardson (2011), this can be achieved simply by reducing the input variance by a factor depending on the ratio of the synaptic to the membrane time constant, since the firing rates depend only little on the synaptic filtering as long as the probability density function of the membrane potential is not altered too much. This, in turn, conveniently allows to apply all the equations based on Gaussian white noise. Testing these ideas by simulations, we found that this simple modification works remarkably well for a wide range of synaptic time constants (cf. Figure 10). Deviations occurred mainly in the supra-rheobase regime where the spiking dynamics is mean-driven. By following the lines of Alijani and Richardson (2011), we derived an expression for the firing rates which explicitly corrects for the synaptic dynamics. This allows to reliably predict the spike rates also in the supra-rheobase regime as long as the synaptic time constant is not too large. Figure 11 furthermore illustrates that the *V*-distributions predicted by the theoretical MF approaches are in good agreement with those from *in vivo* patch-clamp recordings from prefrontal cortex (courtesy of Thomas Hahn, Central Institute of Mental health) where NMDA receptor densities are particularly high (Monaghan and Cotman, 1985).

### 3.5. Extensions and possible improvements

For practical reasons, it might be interesting to see how one can improve the EIF-F(w) approximation, but at the same time keep the computation time requirements as low as possible. Taking into account the empirical probability density of *w* does not improve results beyond its parametric-analytical instantiation through the truncated gamma-function. These theoretical approximations could potentially be further improved by incorporating the implicit *V*-dependency of *w* explicitly. However, these steps are mathematically challenging due to the exponential term in the AdEx equations modeling the action potential upswing. In order to at least test the predictive power of these possible enhancements, one could return to piecewise defined linear models (Hertäg et al., 2012) which still capture most of the physiological features observed in real neurons.

These approaches could also be carried forward to other simple neuron models, as the refractory EIF (rEIF, Badel et al., 2008b), the adaptive LIF model (aLIF, Hertäg et al., 2012) or the AdEx with subthreshold adaptation (i.e., *a* ≠ 0 in Equation 2). In Brunel et al. (2003), the generalized integrate-and-fire neuron (GIF) was addressed which accounts for subthreshold, but not spike-triggered adaptation. The second-order steady-state firing rate corrections obtained from both these approaches could easily be combined (*a* ≠ 0 and *b* ≠ 0) which would allow to incorporate simultaneously both types of adaptation.

The calculations of the static transfer function of the aEIF developed here can potentially provide the basis for a mean-field analysis of network asynchronous states for large networks of model cells whose parameters are derived from fitting real cells, bridging an important gap in the literature. The true diversity of cortical cell types could be captured by such an approach as well, by defining different pools of neurons with different average parameter settings (see e.g., Brunel and Wang, 2001). The tools provided here should therefore help to gain a deeper understanding of the mechanistic relationships among the physiological/biophysical or anatomical properties of the network, its neural dynamics, and the cognitive and behavioral phenomena emerging from the latter.

## 4. Models and methods

### 4.1. The solution of the fokker-planck equation for the aEIF in the long-correlation time limit

By assuming ϵ ≪ 1 (long correlation time limit), the probability distribution and the firing rate can be expanded in powers of ϵ

(36) $$P(V,z)=\sum_{i=0}^{\infty }\epsilon^{i}\cdot P_{i}(V,z),$$

(37) $$\;\nu =\sum_{i=0}^{\infty }\epsilon^{i}\cdot \nu_{i}.$$

In order to derive equations for the *i*-th order correction *P*_*i*_ and ν_*i*_, respectively, the time series are inserted into the time-independent Fokker-Planck equation (Equation 11) and sorted by the power of ϵ. Thereby, the expression $\sqrt{\nu }$ has to be expressed by its Taylor series expansion,

(38) $$\begin{array}{l} \sqrt{\nu }=\sqrt{\sum_{i=0}^{\infty }\epsilon^{i}\cdot \nu_{i}}=\sqrt{\nu_{0}}\sqrt{1+\sum_{i=1}^{\infty }\epsilon^{i}\cdot \frac{\nu_{i}}{\nu_{0}}}\\ \;=\sqrt{\nu_{0}}\sum_{n=0}^{\infty }\left(\begin{array}{c} 1/2\\ n \end{array}\right){\left[\sum_{i=1}^{\infty }\epsilon^{i}\cdot \frac{\nu_{i}}{\nu_{0}}\right]}^{n}. \end{array}$$

Equation (38) can be re-written by

(39) $$\sqrt{\nu }=\sqrt{\nu_{0}}\left(1+\sum_{L=1}^{\infty }\epsilon^{L}\sum_{i=1}^{L}\left(\begin{array}{c} 1/2\\ i \end{array}\right)\frac{1}{\nu_{0}^{i}}\sum_{X\in (A^{i}|\sum X=L)}\prod_{k\in X}\nu_{k}\right)$$

with the set *A* given by *A* = {1, 2, …, *L*}. The set operation *A*^*i*^ is defined as creating a new set of all ordered pairs where an element of *A*^*i*^ is a vector of *i* elements of *A*. Hence, *X* is an *i*-dimensional vector which meets the additional constraint that the sum of its elements is equal *L*. By inserting the time series into the steady-state Fokker-Planck equation (Equation 11), one can derive a general differential equation for the *P*_*i*_(*V*, *z*),

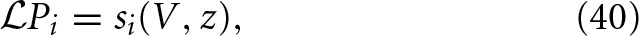



with the operator 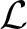 defined by 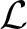
$•=\partial_{V}\;[(f(V)-b\nu_{0})•]-\frac{\sigma^{2}}{2}\partial_{V}^{2}•$ and *s*_*i*_(*V*, *z*) determined order by order by the inhomogeneous terms (see Equation 11). Hence, the general solution *P*_*i*_(*V*, *z*) is directly given by

(41) $$P_{i}(V,z)=\frac{2}{\sigma^{2}}\int_{V}^{V_{\text{up}}}S_{i}(u,z)e^{-\frac{2}{\sigma^{2}}\int_{V}^{u}(f(x)-b\nu_{0})dx}du,$$

with *S*_*i*_ the general antiderivative of *s*_*i*_. A complete description of the problem and the identification of *S*_*i*_(*V*, *z*) requires the definition of the boundary conditions:

(42) $$P(V_{\text{up}},z)={\left[P(V,z)\right]}_{V_{r}^{-}}^{V_{r}^{+}}=0$$

(43) $$\begin{array}{l} \partial_{V}P(V,z){|}_{V=V_{\text{up}}}+\frac{2}{\sigma^{2}}R(z)=\partial_{V}P(V,z){|}_{V_{r}^{-}}^{V_{r}^{+}}\\ \;+\frac{2}{\sigma^{2}}\sum_{i=0}^{\infty }\frac{{\left(-1\right)}^{i}}{i!}{\left(\frac{\epsilon }{\sqrt{\nu }}\right)}^{i}R^{\left(i\right)}(z)=0. \end{array}$$

with *V*^+^_*r*_ and *V*^−^_*r*_ denoting the membrane potential immediately above and below the reset value. The second boundary condition incorporates the jump constraint (spike triggered adaptation defined by adding a constant *b* at each spike). The *i*-th order firing rate correction is then given by the integral of the *i*-th order probability current correction at the upper threshold *V*_up_ over the *z*-space. By explicitly using the above-defined boundary conditions, one can also write,

(44) $$\nu_{i}(t)=\int_{-\infty }^{\infty }R_{i}(z)dz.$$

The determination of the firing rate corrections comes down to the derivation of the *R*_*i*_(*z*). Since *P*(*V*, *z*) is a probability distribution, the integral over the entire space is one and has to be independent of ϵ (Brunel and Latham, 2003). Consequently, one has

(45) $$\;1=\int_{-\infty }^{\infty }dz\int_{-\infty }^{V_{\text{up}}}dVP_{0}(V,z)$$

(46) $$0=\int_{-\infty }^{\infty }dz\int_{-\infty }^{V_{\text{up}}}dVP_{i}(V,z)\;\forall i\geq 1.$$

Furthermore, because of the ϵ − *z* − symmetry of the Fokker-Planck equation and the fact that the firing rate is the integral over the *z*-space from −∞ to ∞, all odd-order contributions to the firing rate are zero (ν_2*n* + 1_ = 0 ∀ *n* ∈ ℕ_0_). With this in mind and defining the inverse operator 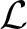
^−1^ = *J* · *K* by

(47) $$\;J•=\frac{2}{\sigma^{2}}\int_{V}^{V_{\text{up}}}du\;e^{-\frac{2}{\sigma^{2}}\int_{V}^{u}(f(x)-b\nu_{0})dx}•,$$

(48) $$K•=\int_{-\infty }^{V}du\;•,$$

the zeroth and second-order firing rate corrections can be derived. Since 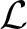
*P*_0_(*V*, *z*) = 0, one can directly write

(49) $$\begin{array}{l} P_{0}(V,z)=Q_{0}(V)R_{0}(z)=\frac{2R_{0}(z)}{\sigma^{2}}\overset{V_{\text{up}}}{\underset{V}{\int }}e^{-\frac{2}{\sigma^{2}}\overset{u}{\underset{V}{\int }}[f(x)-b\nu_{0}]dx}\\ \;\Theta (V-V_{r})du \end{array}$$

with Θ(*x*) the Heaviside step function. The unknown function *R*_0_(*z*) will be determined at higher orders. Note that Equation (49) fulfills all boundary conditions (cf. with Equations 42, 43). Since 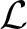
$P_{1}=b\sqrt{\nu_{0}}zR_{0}\partial_{V}Q_{0}+\sqrt{\nu_{0}}R_{0}{}^{\text{′}}Q_{0}$, for the first order correction *P*_1_(*V*, *z*), one obtains by incorporating the boundary conditions

(50) $$\begin{array}{l} P_{1}(V,z)=\left(R_{1}(z)-\frac{R_{0}^{\prime }(z)}{\sqrt{\nu_{0}}}\right)Q_{0}(V)+b\sqrt{\nu_{0}}zR_{0}(z)JQ_{0}(V)\\ \;+\;\sqrt{\nu_{0}}R_{0}^{\prime }(z)JKQ_{0}(V) \end{array}$$

Deriving *P*_2_(*V*, *z*) in the same manner yields

(51) $$\begin{array}{l} P_{2}(V,z)=\left(R_{2}-\frac{R_{0}^{\prime }}{\sqrt{\nu_{0}}}+\frac{R_{0}^{\prime \prime }}{2\nu_{0}}\right)Q_{0}+b\nu_{2}R_{0}JQ_{0}\\ \;+b\sqrt{\nu_{0}}zJ(b\sqrt{\nu_{0}}zR_{0}JQ_{0}+\sqrt{\nu_{0}}R_{0}^{\prime }JKQ_{0}+\ldots \\ \;\ldots +\left[R_{1}-\frac{R_{0}^{\prime }}{\sqrt{\nu_{0}}}\right]Q_{0})+{\left(zR_{0}\right)}^{\prime }JKQ_{0}\\ \;+\sqrt{\nu_{0}}(b\sqrt{\nu_{0}}{\left(zR_{0}\right)}^{\prime }JKJQ_{0}+\ldots \\ \;\ldots +\sqrt{\nu_{0}}R_{0}^{\prime \prime }JKJKQ_{0}+\;\left[R_{1}^{\prime }-\frac{R_{0}^{\prime \prime }}{\sqrt{\nu_{0}}}\right]JKQ_{0}) \end{array}$$

The second boundary condition (43) leads to

(52) $$\begin{array}{l} -\frac{\sigma^{2}}{2}\partial_{V}P_{2}(V,z){|}_{V=V_{\text{up}}}=R_{2}-\frac{R_{1}^{\prime }}{\sqrt{\nu_{0}}}+\frac{R_{0}^{\prime \prime }}{2\nu_{0}}+{\left(zR_{0}\right)}^{\prime }KQ_{0}(V_{\text{up}})\\ \;+b\nu_{0}{\left(zR_{0}\right)}^{\prime }KJQ_{0}(V_{\text{up}})-\ldots \\ \;\ldots +\nu_{0}R_{0}^{\prime \prime }KJKQ_{0}(V_{\text{up}})\\ \;+\;\frac{R_{1}^{\prime }}{\sqrt{\nu_{0}}}-\frac{R_{0}^{\prime \prime }}{\nu_{0}}=R_{2}. \end{array}$$

Hence, in order to comply with the boundary conditions for *w* and *V*, *R*_0_(*z*) needs to satisfy

(53) $$\begin{array}{l} 0=\left[\frac{1}{\nu_{0}}+b\nu_{0}KJQ_{0}(V_{\text{up}})\right]{\left(zR_{0}\right)}^{\prime }\\ \;+\left[\nu_{0}KJKQ_{0}(V_{\text{up}})-\frac{1}{2\nu_{0}}\right]R_{0}^{\prime \prime } \end{array}$$

whose solution is

(54) $$R_{0}(z)=\frac{\nu_{0}}{\sqrt{2\pi {\langle z^{2}\rangle }_{0}}}\text{exp}\left(-\frac{z^{2}}{2{\langle z^{2}\rangle }_{0}}\right),$$

with

(55) $${\langle z^{2}\rangle }_{0}=\frac{\left(\nu_{0}KJKQ_{0}(V_{\text{up}})-\frac{1}{2\nu_{0}}\right)}{\left(\frac{1}{\nu_{0}}+b\nu_{0}KJQ_{0}(V_{\text{up}})\right)}.$$

The second order correction to the firing rate ν_2_ is now given by integrating *P*_2_ over the *z* − *V* − space and applying the condition (46):

(56) $$\begin{array}{l} \nu_{2}\left(KJKQ_{0}(V_{\text{up}})-\frac{1}{2\nu_{0}^{2}}\right)=b{\langle z^{2}\rangle }_{0}[\nu_{0}KJ^{2}KQ_{0}(V_{\text{up}})\\ \;-b\nu_{0}{\langle z^{2}\rangle }_{0}KJ^{2}Q_{0}(V_{\text{up}})+\ldots \\ \;\ldots -\frac{KJQ_{0}(V_{\text{up}})}{\sqrt{\nu_{0}}}\int_{-\infty }^{\infty }zR_{1}dz\\ \;-KJQ_{0}(V_{\text{up}})]. \end{array}$$

The final step is to determine the integral $\int_{-\infty }^{\infty }zR_{1}dz$ by integrating

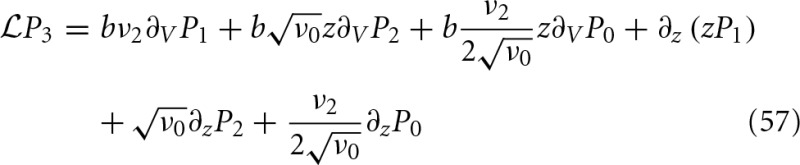



once from −∞ to *V*_up_ that yields

(58) $$\begin{array}{l} \frac{R_{2}^{\prime }}{\sqrt{\nu_{0}}}-\frac{\nu_{2}}{2\nu_{0}^{3/2}}R_{0}^{\prime }-\frac{R_{1}^{\prime \prime }}{2\nu_{0}}+\frac{R_{1}^{�}}{6\nu_{0}^{3/2}}=\partial_{z}\left[zKP_{1}(V_{\text{up}})\right]\\ \;+\sqrt{\nu_{0}}\partial_{z}KP_{2}(V_{\text{up}})\\ \;+\frac{\nu_{2}}{2\sqrt{\nu_{0}}}\partial_{z}KP_{0}(V_{\text{up}}),\; \end{array}$$

which in turn leads to a differential equation for the unknown function *R*_1_(*z*):

(59) $$\begin{array}{l} \text{ ​​}-\left[\nu_{0}KJKQ_{0}(V_{\text{up}})-\frac{1}{2\nu_{0}}\right]R_{1}^{\prime \prime }-\left[\frac{1}{\nu_{0}}+b\nu_{0}KJQ_{0}(V_{\text{up}})\right]{\left(zR_{1}\right)}^{\prime }\\ =R_{0}^{\prime }\left[\sqrt{\nu_{0}}b\nu_{2}KJQ_{0}(V_{\text{up}})+\frac{\nu_{2}}{\nu_{0}^{3/2}}\right]+\frac{\frac{1}{\nu_{0}}+b\nu_{0}KJQ}{\sqrt{\nu_{0}}{\langle z^{2}\rangle }_{0}}{\left(z^{2}R_{0}\right)}^{\prime }\ldots \\ \;\ldots +\;\frac{R_{0}^{�}}{3\nu_{0}^{3/2}}+{\left(z^{2}R_{0}\right)}^{\prime }\sqrt{\nu_{0}}KJ[bQ_{0}(V_{\text{up}})-\frac{KQ_{0}(V_{\text{up}})}{{\langle z^{2}\rangle }_{0}}\\ \;+\;b^{2}\nu_{0}JQ_{0}(V_{\text{up}})-\frac{b\nu_{0}JKQ_{0}(V_{\text{up}})}{{\langle z^{2}\rangle }_{0}}]\\ \;\ldots +R_{0}^{�}\sqrt{\nu_{0}}KJK[\nu_{0}JKQ_{0}(V_{\text{up}})-{\langle z^{2}\rangle }_{0}Q_{0}(V_{\text{up}})\\ \;-b{\langle z^{2}\rangle }_{0}\nu_{0}JQ_{0}(V_{\text{up}})-2Q_{0}(V_{\text{up}})],\text{​​​​​} \end{array}$$

The solution of Equation (59) is given by *R*_0_ multiplied with a polynomial containing only odd-order contributions. The integral $\int_{-\infty }^{\infty }zR_{1}dz$ can be conveniently obtained by integrating (59) twice over *z*:

(60) $$\begin{array}{l} -\int_{-\infty }^{\infty }zR_{1}dz=\frac{{\langle z^{2}\rangle }_{0}^{2}\sqrt{\nu_{0}}KJ}{KJKQ_{0}(V_{\text{up}})-\frac{1}{2\nu_{0}^{2}}}[bQ_{0}(V_{\text{up}})-\frac{KQ_{0}(V_{\text{up}})}{{\langle z^{2}\rangle }_{0}}\\ \;+\;b^{2}\nu_{0}JQ_{0}(V_{\text{up}})-\frac{b\nu_{0}JKQ_{0}(V_{\text{up}})}{{\langle z^{2}\rangle }_{0}}]+\\ \;\ldots +\frac{{\langle z^{2}\rangle }_{0}}{KJKQ_{0}(V_{\text{up}})-\frac{1}{2\nu_{0}^{2}}}\\ \;\left[\sqrt{\nu_{0}}b\nu_{2}KJQ_{0}(V_{\text{up}})+\frac{\nu_{2}}{\nu_{0}^{3/2}}\right]-\sqrt{\nu_{0}}. \end{array}$$

Equations (56) and (60) determine the second order correction ν_2_.

### 4.2. The derivation of the variance of *w*

Along the lines of Takács (1955), we introduce a random variable η(*t*) that denotes the sum of the values of *w* in the time interval (0, *t*),

(61) $$\eta (t)=\sum_{0\leq qt_{n}\leq qt}w(t-t_{n}).$$

with *w*(*t* − *t*_*n*_) defined as

(62) $$w(t-t_{n})=\{\begin{array}{c} \begin{array}{cc} b/g_{L}\cdot e^{-(t-t_{n})/\tau_{w}} & \text{if }(t-t_{n})\geq 0\\ 0 & \text{if }(t-t_{n})<0 \end{array} \end{array}$$

In order to derive the moments of the stochastic process, we consider the value of the sum of the signals directly before the beginning of the *n*-th event,

(63) $$\eta (t_{n}-0)\equiv \eta_{n+1}=\left(\eta_{n}+\frac{b}{g_{L}}\right)\cdot e^{-\Delta t/\tau_{w}},$$

with Δ*t* the n-th interspike interval. Any moment can be derived directly from this relation by the recurrence formula (Takács, 1955),

(64) $$\begin{array}{l} E\left\{\eta_{n+1}^{k}\right\}=E\left\{e^{-k\Delta t/\tau_{w}}\right\}E\left\{{\left(\eta_{n}+b/g_{L}\right)}^{k}\right\}\\ \;=\beta_{k}\frac{b}{g_{L}}\sum_{j=0}^{k}\left(\begin{array}{c} k\\ j \end{array}\right)E\left\{\eta_{n}^{j}\right\} \end{array}$$

(65) $$\begin{array}{l} \;\beta_{k}=E\left\{e^{-k\Delta t/\tau_{w}}\right\}=\int_{0}^{\infty }e^{-k\Delta t/\tau_{w}}dG(x)\\ \text{ with }P(\Delta t\leq x)=G(x) \end{array}$$

Here, *E*{•} denotes the expectancy value, and *G* represents the cumulative distribution for the interspike intervals. If the limit $\underset{n\to \infty }{\lim}E\{\eta_{n}^{k}\}=E_{k}$ exists for all *k*, then they can be obtained by Takács (1955)

(66) $$E_{k}=\beta_{k}\frac{b}{g_{L}}\sum_{j=0}^{k}\left(\begin{array}{c} k\\ j \end{array}\right)E_{j}.$$

The first two moments *E*_1_ and *E*_2_ are then given by

(67) $$E_{1}=\frac{b\beta_{1}}{g_{L}\cdot (1-\beta_{1})}$$

(68) $$E_{2}=\frac{b^{2}(1+\beta_{1})}{g_{L}^{2}(1-\beta_{1})(1-\beta_{2})}\beta_{2}.$$

If a limiting distribution for *w* exists (see p. 369, Theorem 2 in Takács, 1955), its *j*-th moment E^*^_j_ can be derived by

(69) $$E_{j}^{*}=\frac{E_{j}\tau_{w}\nu }{\beta_{j}}\frac{1-\beta_{j}}{j}$$

with *E*_*j*_ given by Equation (66) and ν the firing rate of the neuron considered. The variance of the stochastic process is defined by the first two moments and yields

(70) $$\sigma_{w}^{2}=E_{2}^{*}-{\left(E_{1}^{*}\right)}^{2}=\frac{b^{2}\tau_{w}\nu }{2\cdot g_{L}^{2}}\left[\frac{1+\beta_{1}}{1-\beta_{1}}-2\tau_{w}\nu \right],$$

with β_1_ the Laplace transform of the ISI-distribution at 1/τ_*w*_, and E^*^_1_ the mean value of *w*,

(71) $$<w>=\frac{b\tau_{w}\nu }{g_{L}}.$$

We assume that the ISI distribution is well approximated by a Gamma-probability density function. In order to compute β_1_, the distributional properties have to be parameterized by its mean μ_ISI_ directly given by the inverse of the firing rate ν and the variance σ^2^_ISI_. The moments can be theoretically derived by the following recurrence relation (see Tuckwell, 1988),

(72) $$\frac{\sigma^{2}}{2}\frac{d^{2}\mu_{k}}{dx^{2}}+f(x)\frac{d\mu_{k}}{dx}=-k\tau \mu_{k-1}$$

with *x* = *V*_*r*_ and *f*(*x*) defined by

(73) $$f(x)=E-x+\Delta_{T}\cdot e^{\frac{x-V_{T}}{\Delta_{T}}}$$

with the effective potential $E=E_{L}+\mu -\frac{b\tau_{w}\nu }{gL}$. The variance σ^2^_ISI_ is directly obtained by the 1st and 2nd moments which are determined by the solution of the differential Equation (72),

(74) $$\begin{array}{l} \sigma_{\text{ISI}}^{2}=\mu_{2}-\mu_{1}^{2}=\mu_{1}^{2}+\frac{8\tau^{2}}{\sigma_{0}^{4}}\int_{V_{r}}^{\infty }dV\;e^{-\frac{2}{\sigma_{0}^{2}}F(V)}\\ \;\int_{-\infty }^{V}du\;e^{-\frac{2}{\sigma_{0}^{2}}F(u)}{\left[\int_{-\infty }^{u}dy\;e^{\frac{2}{\sigma_{0}^{2}}F(y)}\;\right]}^{2}-\mu_{1}^{2} \end{array}$$

(75) $$=\frac{8\tau^{2}}{\sigma_{0}^{4}}\text{​​}\int_{V_{r}}^{\infty }dV\;e^{-\frac{2}{\sigma_{0}^{2}}F(V)}\text{​​}\int_{-\infty }^{V}\text{​}du\;e^{-\frac{2}{\sigma_{0}^{2}}F(u)}\text{​​}{\left[\int_{-\infty }^{u}dy\;e^{\frac{2}{\sigma_{0}^{2}}F(y)}\;\right]}^{2}$$

with $F(x)=E\cdot x-\frac{x^{2}}{2}+\Delta_{T}e^{(x-V_{T})/\Delta_{T}}$ the general antiderivative of *f*(*x*). By parameterizing the constants *k* and Θ of the Gamma-distribution *P*(*x*; *k*, θ) by

(76) $$k={\left(\nu^{2}\sigma_{\text{ISI}}^{2}\right)}^{-1},$$

(77) $$\theta =\nu \sigma_{\text{ISI}}^{2},$$

the Laplace transform of the ISI-distribution at 1/τ_*w*_ yields

(78) $$\begin{array}{l} \beta_{1}=\int_{0}^{\infty }e^{-\frac{x}{\tau_{w}}}\cdot P(x;k,\theta )dx=\frac{1}{\theta^{k}\Gamma (k)}\int_{0}^{\infty }x^{k-1}\cdot e^{-\left(\frac{1}{\tau_{w}}+\frac{1}{\theta }\right)\cdot x}dx\\ \;=\frac{1}{\theta^{k}\Gamma (k)}{\left(\frac{\tau_{w}\theta }{\theta +\tau_{w}}\right)}^{k}\int_{0}^{\infty }z^{k-1}e^{-z}dz={\left(\frac{\tau_{w}}{\theta +\tau_{w}}\right)}^{k}. \end{array}$$

### 4.3. The derivation of the second-order firing rate correction for synaptic kinetics

In order to compute the firing rate correction for the aEIF model under synaptic dynamics, we have to consider the EIF with averaged *w*-component and synaptic noise obeying the following differential equation

(79) $$\begin{array}{l} \tau_{m}\cdot \frac{dV}{dt}=-(V-E_{L})+\Delta_{T}\cdot e^{\left(\frac{V-V_{T}}{\Delta_{T}}\right)}+I/g_{L}-b\tau_{w}\nu /g_{L}\\ \;=f(V)+I/g_{L} \end{array}$$

(80) $$\;\tau_{s}\cdot \frac{dI}{dt}=-I+\sqrt{\tau_{m}}\sigma \eta (t)$$

When *V* reaches “infinity,” the membrane potential is reset to *V*_*r*_. The FP equation with the boundary conditions explicitly included, is given by Alijani and Richardson (2011)

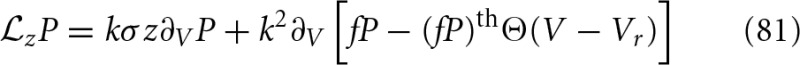



with $k=\sqrt{\tau_{s}/\tau_{m}}$, (*fP*)^th^ denoting *fP* evaluated in the limit *V* → ∞ and

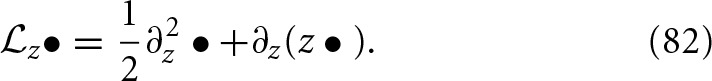



By using the respective continuity equation and the FP-equation at the same time, one can derive the following relation for the firing rate (Alijani and Richardson, 2011)

(83) $$\begin{array}{l} \nu \tau_{m}\Theta (V-V_{r})=f\{1\}-\sigma^{2}\partial_{V}\{z^{2}\}\\ \;-\;\sigma k\partial_{V}\left[f\{z\}-{\left(f\{z\}\right)}^{\text{th}}\theta (V-V_{r})\right], \end{array}$$

where $\{•\}=\int_{-\infty }^{\infty }P(V,z)•\;dz$. By assuming *k* ≪ 1, the probability distribution and the firing rate can be expanded in powers of *k*. Consequently, the zeroth order which is already known by the Gaussian white noise case can be written by

(84) $$P_{0}=\Phi_{0}\cdot Q_{0}$$

with Φ_0_ directly given by the operator 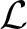
_*z*_ and the boundary conditions:

(85) $$\Phi_{0}=\sqrt{\pi }\cdot e^{-z^{2}}.$$

By making use of the relations

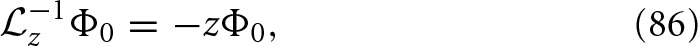

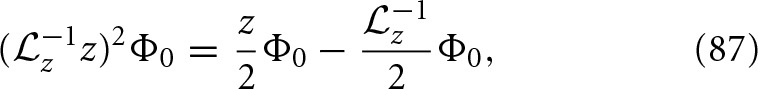





and evaluating Equation (81) at first and second order, one obtains

(88) $$\;P_{1}=-\sigma \partial_{V}Q_{0}z\Phi_{0},$$

(89) $$P_{2}=\Phi_{0}Q_{2}+\frac{\sigma^{2}}{2}z^{2}\Phi_{0}\partial_{V}^{2}Q_{0}.$$

The derivation of

(90) $${\left\{1\right\}}_{2}=2{\left\{z^{2}\right\}}_{2}-\frac{\sigma^{2}}{2}\partial_{V}^{2}Q_{0},$$

(91) $${\left\{z\right\}}_{1}=-\frac{\sigma }{2}\partial_{V}Q_{0}$$

allows to deduce a differential equation for ν_2_ [see Equation (83)] (Alijani and Richardson, 2011):

(92) $$\begin{array}{l} \tau_{m}\nu_{2}\Theta (V-V_{r})=(2f-\sigma^{2}\partial_{V})\{z^{2}\}+\frac{\sigma^{2}}{2}\partial_{V}f\partial_{V}Q_{0}\\ \;-\frac{\sigma^{2}}{2}\partial_{V}{\left(f\partial_{V}Q_{0}\right)}^{\text{th}}\Theta (V-V_{r}). \end{array}$$

This can be rewritten into (Alijani and Richardson, 2011)

(93) $$\begin{array}{l} (2f-\sigma^{2}\partial_{V})\{z^{2}\}=\tau_{m}\nu_{2}\Theta (V-V_{r})-\frac{\sigma^{2}}{2}\partial_{V}f\partial_{V}Q_{0}\\ \;-\frac{\sigma^{2}\nu_{0}\tau_{m}}{2\Delta_{T}}\delta (V-V_{r}). \end{array}$$

By making use of the derivative of *Q*_0_, one can simplify the previous relation by

(94) $$\begin{array}{l} (2f-\sigma^{2}\partial_{V})\left(\{z^{2}\}-\frac{\nu_{2}}{2\nu_{0}}Q_{0}+\frac{\sigma^{2}}{4\Delta_{T}}\partial_{V}Q_{0}\right)\\ \;=-\frac{\sigma^{2}}{4}\left[\frac{Q_{0}}{\Delta_{T}}+\partial_{V}Q_{0}\right]\partial_{V}f. \end{array}$$

Solving the differential equation for {*z*^2^} yields

(95) $$\begin{array}{l} \{z^{2}\}=\frac{\nu_{2}}{2\nu_{0}}Q_{0}-\frac{\sigma^{2}}{4\Delta_{T}}\partial_{V}Q_{0}\\ \;-\frac{1}{2}\int_{V}^{\infty }du\;e^{-\frac{2}{\sigma^{2}}\int_{V}^{u}f(x)dx}\left[\frac{Q_{0}}{\Delta_{T}}+\partial_{u}Q_{0}\right]\partial_{u}f. \end{array}$$

Taking the integral over *V* from −∞ to ∞ yields an expression for the second-order firing rate correction

(96) $$\nu_{2}=\nu_{0}\int_{-\infty }^{\infty }dV\;\int_{V}^{\infty }du\;e^{-\frac{2}{\sigma^{2}}\int_{V}^{u}f(x)dx}\left[\frac{Q_{0}}{\Delta_{T}}+\partial_{u}Q_{0}\right]\partial_{u}f.$$

Hence, the firing rate in the colored noise case can be computed by ν_CN_ = ν_0_ + *k*^2^ν_2, CN_ with ν_2, CN_ given by (96). By assuming that the *w*-distribution in the case of synaptic dynamics can still be replaced by a Gamma function parametrized by mean and variance of the adaptation current, one obtains Equation (34).

### 4.4. Simulation and analysis details

All simulations were run on a 2.4 GHz Intel(R) Xeon(R) CPU E5620. The single-cell simulations for the purpose of evaluating the prediction power of the theoretical approaches were 50–100 s long, while the first 5 s were discarded and only the remaining part was applied to estimate the steady-state firing rates. The time increment was 0.05 ms. Simulations with smaller time steps did not change the results. For evaluating the subthreshold voltage fluctuations spikes were cut out in a window of ±10 ms around an action potential. All implementations were written in Matlab and C. Matlab-based code and the data sets used in this study can be obtained from the authors on request & and will be made publicly available at *www.bccn-heidelberg-mannheim.de*.

### Conflict of interest statement

The authors declare that the research was conducted in the absence of any commercial or financial relationships that could be construed as a potential conflict of interest.
